# Mitochondrial complex I activity in microglia sustains neuroinflammation

**DOI:** 10.1038/s41586-024-07167-9

**Published:** 2024-03-13

**Authors:** L. Peruzzotti-Jametti, C. M. Willis, G. Krzak, R. Hamel, L. Pirvan, R.-B. Ionescu, J. A. Reisz, H. A. Prag, M. E. Garcia-Segura, V. Wu, Y. Xiang, B. Barlas, A. M. Casey, A. M. R. van den Bosch, A. M. Nicaise, L. Roth, G. R. Bates, H. Huang, P. Prasad, A. E. Vincent, C. Frezza, C. Viscomi, G. Balmus, Z. Takats, J. C. Marioni, A. D’Alessandro, M. P. Murphy, I. Mohorianu, S. Pluchino

**Affiliations:** 1 Department of Clinical Neurosciences and NIHR Biomedical Research Centre, University of Cambridge, Cambridge, UK; 2 Department of Metabolism, Digestion and Reproduction, Imperial College London, London, UK; 3 Wellcome-MRC Cambridge Stem Cell Institute, University of Cambridge, Cambridge, UK; 4 Department of Biochemistry and Molecular Genetics, University of Colorado School of Medicine, Aurora, CO USA; 5 MRC Mitochondrial Biology Unit, University of Cambridge, Cambridge Biomedical Campus, Cambridge, UK; 6 UK Dementia Research Institute, University of Cambridge, Cambridge, UK; 7 Wellcome Centre for Mitochondrial Research, Translational and Clinical Research Institute, Faculty of Medical Sciences, Newcastle University, Newcastle upon Tyne, UK; 8 University Hospital Cologne, Cologne, Germany; 9 University of Padua, Padova, Italy; 10 Department of Molecular Neuroscience, Transylvanian Institute of Neuroscience, Cluj-Napoca, Romania; 11 European Molecular Biology Laboratory, European Bioinformatics Institute, EMBL-EBI, Wellcome Genome Campus, Hinxton, UK

**Keywords:** Neuroimmunology, Microglia

## Abstract

Sustained smouldering, or low-grade activation, of myeloid cells is a common hallmark of several chronic neurological diseases, including multiple sclerosis^1^. Distinct metabolic and mitochondrial features guide the activation and the diverse functional states of myeloid cells^2^. However, how these metabolic features act to perpetuate inflammation of the central nervous system is unclear. Here, using a multiomics approach, we identify a molecular signature that sustains the activation of microglia through mitochondrial complex I activity driving reverse electron transport and the production of reactive oxygen species. Mechanistically, blocking complex I in pro-inflammatory microglia protects the central nervous system against neurotoxic damage and improves functional outcomes in an animal disease model in vivo. Complex I activity in microglia is a potential therapeutic target to foster neuroprotection in chronic inflammatory disorders of the central nervous system^3^.

## Abstract (web summary)

Blocking mitochondrial complex I in pro-inflammatory microglia protects the central nervous system against neurotoxic damage and improves functional outcomes in vivo in an animal disease model.

## Main

In multiple sclerosis (MS), chronic active, slowly expanding, smouldering lesions characterized by the accumulation of myeloid cells at the lesion edge are associated with brain atrophy and predict the accumulation of irreversible disability, which in turn drives disease progression^4–6^. In these lesions, persistently activated myeloid cells are a continuous source of neurotoxic factors, including tumour necrosis factor (TNF), interleukin-1β (IL-1β), nitric oxide (NO) and reactive oxygen species (ROS), causing remyelination failure and secondary neuronal/axonal damage^7^. In MS-like disease models, axonal injury is followed by a compensatory response, whereby mitochondrial content and activity increases in demyelinated axons to promote neuroprotection^8,9^. On the contrary, deficits in neuronal mitochondrial complexes and energy metabolism have been associated with persistent axonal damage, grey-matter atrophy and MS disease progression^10,11^.

Mitochondrial respiratory complexes and metabolites are also known to control myeloid immune responses^12,13^. Previous in vitro studies have shown that, under inflammatory conditions, elevated intracellular succinate levels in myeloid cells promote a switch from the normal forward electron transport along the respiratory chain to reverse electron transport (RET) through mitochondrial complex I (C)I^12^. This mechanism, which requires a high proton motive force^14^, effectively repurposes mitochondria away from the production of adenosine triphosphate (ATP) towards the generation of superoxide that goes on to form hydrogen peroxide and other ROS, together called mitochondrial ROS (mtROS)^12^. Inhibition of succinate dehydrogenase (also known as mitochondrial complex II (CII)) by the reversible inhibitors itaconate or malonate limits RET-induced mtROS production and promotes anti-inflammatory effects in myeloid cells in vitro^15,16^. Similarly, blocking CII or CI activity protects against RET-mediated mtROS damage during reperfusion in the infarcted heart in vivo^17,18^. However, the role of mitochondrial complexes in perpetuating the activation of microglia in the context of smouldering inflammatory central nervous system (CNS) diseases remains largely unexplored.

**Extended Data Fig. 1:**
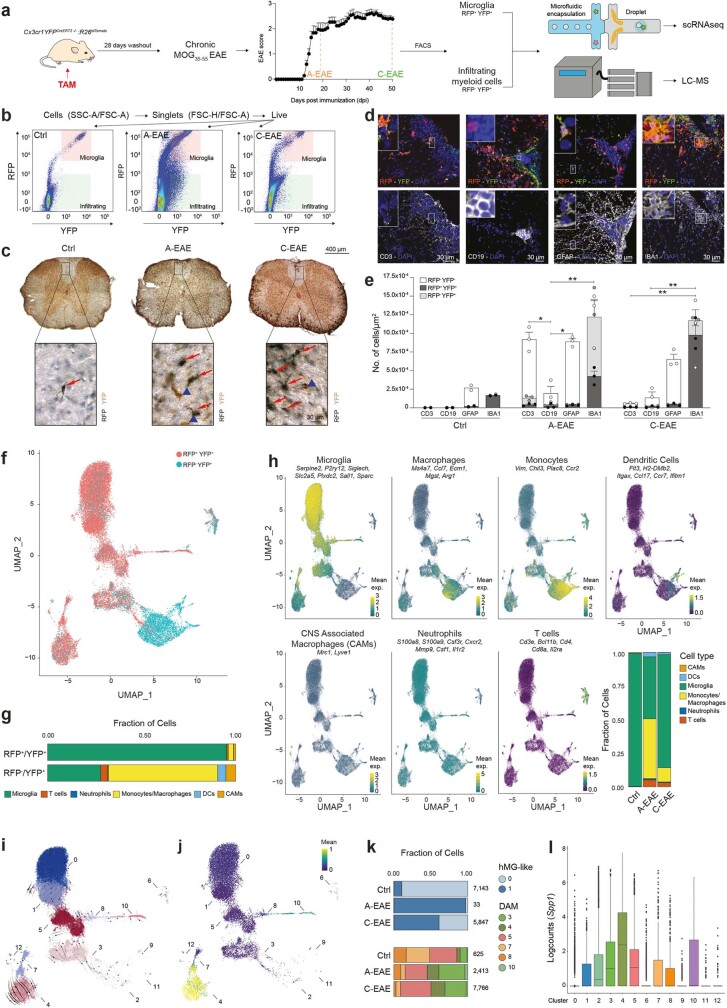
Characterization of microglia and infiltrating myeloid cells in the *Cx3cr1*YFP^CreERT2^:R26^tdTomato^ fate mapping mouse. **a**, Experimental design for the ex vivo scRNAseq and LC-MS studies. TAM: tamoxifen. **b**, Representative flow cytometry plots of RFP^+^ YFP^+^ microglia and RFP^-^ YFP^+^ infiltrating myeloid cells in Ctrl, A-EAE, and C-EAE mice. **c**, Representative immunohistochemistry of RFP^+^ YFP^+^ microglia (black, red arrows) and RFP^-^YFP^+^ infiltrating myeloid cells (brown, blue arrowheads) in Ctrl, A-EAE, and C-EAE mice. **d**, **e**, Representative (**d**) images and (**e**) quantification of the number of CD3^+^, CD19^+^, GFAP^+^ and IBA1^+^ cells that are RFP^−^ YFP^−^, RFP^+^ YFP^+^, or RFP^-^ YFP^+^ in Ctrl, A-EAE, and C-EAE mice (n = 2, 3, 3 replicates per group; mean ± SEM; *P < 0.05, **P < 0.01; one-way ANOVA; Tukey comparing total number of cells). **f**, UMAP plots from the scRNAseq dataset of cells isolated from the *Cx3cr1*YFP^CreERT2^:R26^tdTomato^ mice (see Fig. 1a). Cells (dots) are coloured as RFP^+^ YFP^+^ microglia or RFP^−^ YFP^+^ myeloid cells. **g**, Bar plot showing the fraction of RFP^+^ YFP^+^ or RFP^−^ YFP^+^ cells expressing core signature genes of the different cell types. We found that nearly the totality of RFP^+^ YFP^+^ cells were transcriptionally microglia. Instead, the majority of RFP^-^ YFP^+^ cells were transcriptionally infiltrating myeloid cells with only a minority (<25%) expressing microglial marker genes, as described in Jordao et al. ^19^ and Hamel et al.^20^. **h**, UMAP plots and quantification of cells expressing core signature genes for microglia, macrophages, monocytes, dendritic cells, CNS associated macrophages (CAMs), neutrophils, and T cells. Cell types were manually assigned based on cluster gene markers and cells are coloured by their mean expression. **i**, **j**, **k**, Unsupervised clustering analysis focused on subpopulations of cells expressing core signature genes for microglia only with (**i**) RNA directionality, (**j**) pseudotime, and (**k**) fraction of cells with a transcriptional signature reminiscent of homeostatic microglia (hMG-like) or disease associated microglia (DAM) in each EAE stage (numbers on the side are total number of cells). **l**, *Spp1* expression in cells isolated ex vivo from *Cx3cr1*YFP^CreERT2^:R26^tdTomato^ Ctrl and EAE mice. Box plots of the log2-transformed counts of *Spp1* showing its expression in all clusters (median and quartiles from 22,148 cells). Source Data

To investigate the molecular mechanisms through which microglia and CNS-infiltrating myeloid cells cooperate to sustain CNS inflammation, we used ex vivo single-cell RNA-sequencing (scRNA-seq) and liquid chromatography–mass spectrometry (LC–MS)-based analyses of *Cx3cr1-YFP*^*creERT2*^*R26*^*tdTomato*^ fate-mapping mice^19,20^, which were immunized with myelin oligodendrocyte glycoprotein peptide 35–55 (MOG_35–55_) to induce experimental autoimmune encephalomyelitis (EAE), a model of MS-like disease (Extended Data Fig. 1). RFP^+^YFP^+^ cells (microglia) and RFP^−^YFP^+^ cells (predominantly consisting of infiltrating myeloid cells^19,20^) were isolated using fluorescence-activated cell sorting (FACS) from the spinal cord of EAE mice in the acute EAE (A-EAE; 3 days after disease onset) and chronic EAE (C-EAE; 50 days after immunization) disease stages. Non-immunized *Cx3cr1-YFP*^*creERT2*^*R26*^*tdTomato*^ mice were used as healthy controls.

**Fig. 1:**
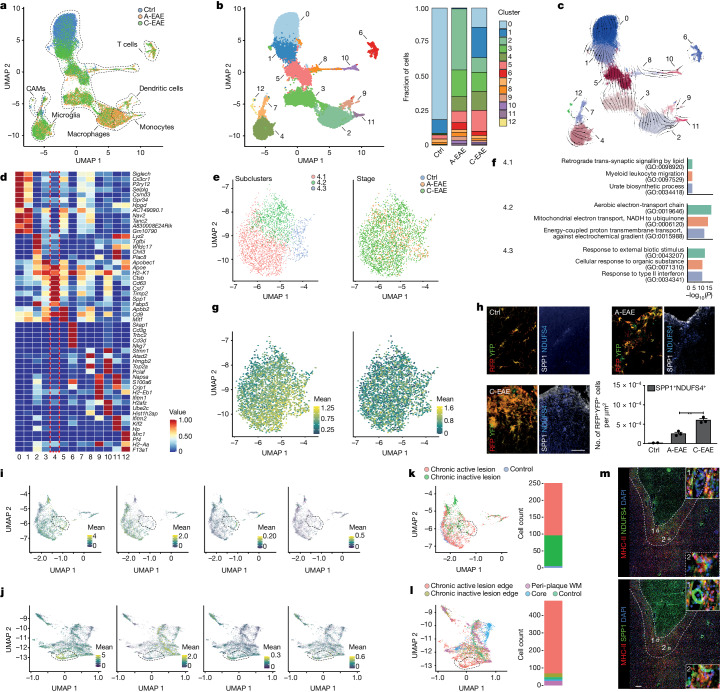
Microglia show increased mitochondrial CI expression during EAE. **a**, scRNA-seq uniform manifold approximation and projection (UMAP) plot obtained from 22,148 cells coloured by EAE stage (6,205 (control), 3,648 (A-EAE), 12,295 (C-EAE)) and fraction of cell types. CAMs, CNS-associated macrophages. **b**, UMAP plot coloured by clusters and fraction of cells per EAE stage. **c**, Streamline plot of RNA velocity underlining RNA expression changes within and across different clusters. The arrows indicate the directionality of transcriptional changes. The thickness is proportional to the velocity (that is, amplitude of changes). **d**, Grouped heat map of the top DEGs for the clusters (plus *Siglech* and *Cx3cr1*). The dotted red box highlights DAM cluster 4. **e**, UMAP plots of the unsupervised subcluster analysis of DAM cluster 4 coloured by subcluster (left) and EAE stage (right). **f**, The top GO terms (by fold enrichment) of DAM cluster 4 subclusters. **g**, UMAP analysis of DAM cluster 4 subclusters coloured by the mean counts of mitochondrial CI (left) and CII (right). **h**, Representative confocal imaging and quantification of EAE lesions, showing the number of SPP1^+^ cells expressing the NADH ubiquinone oxidoreductase iron-sulfur protein 4 (NDUFS4). From left to right, *n* = 2, 3 and 3 replicates per group. Data are mean ± s.e.m. Statistical analysis was performed using one-way analysis of variance (ANOVA) with Fisher’s least significant difference (LSD) test; **P < 0.01. Scale bar, 50 μm. **i**,**j**, Expression UMAPs of *SPP1*, *P2RY12*, and mitochondrial CI and CII genes in human MAMS from two published studies of patients with MS (ref. ^27^ (**i**) and ref. ^28^ (**j**)). **k**,**l**, UMAP and bar chart showing the localization of MAMS in MS lesions and controls from ref. ^27^ (**k**) and ref. ^28^ (**l**). **m**, Representative immunofluorescence (out of three) showing rim-specific expression (dotted lines) of NDUFS4^+^ and SPP1^+^ myeloid cells (MHC-II^+^) in consecutive sections of a chronic active lesion from the secondary progressive MS brain. Scale bar, 60 μm. Source Data

scRNA-seq data showed a prevalence of infiltrating myeloid cells in A-EAE mice, while microglia were predominant in control and C-EAE mice (Fig. 1a and Extended Data Fig. 1). Unsupervised clustering analysis of the integrated dataset identified 13 cell clusters between control, A-EAE and C-EAE mice (Fig. 1b). Clusters 0 and 1 comprised cells with a transcriptional signature reminiscent of homeostatic microglia (hMG-like) and were found mostly in control mice, in which they constituted 91% of all isolated cells. These two clusters differed in the expression of specific homeostatic genes (for example, *Siglech, P2ry12* and *Cx3cr1*, higher in cluster 0) and by the relatively increased expression of *AC149090.1*, a gene encoding a phospholipid decarboxylase that is involved in lipid metabolism, in cluster 1. This is consistent with evidence supporting differential cellular transcriptional states of microglia^21^. The proportion of hMG-like cells decreased to less than 1% of all isolated cells in A-EAE mice, and subsequently increased to 36% of cells in C-EAE mice, therefore suggesting a partial return to homeostasis in the chronic stage of disease^7^ (Fig. 1b). Our approach also identified several clusters (that is, clusters 3, 4, 5, 7, 8 and 10) of disease-associated microglia (DAMs)^22^ that were nearly absent in control mice, increased in A-EAE mice and persisted in C-EAE mice (8%, 46% and 48% of all isolated cells, respectively) (Fig. 1b).

Given the potential pathogenic role of persistent DAM activity in chronic CNS diseases^23^, we performed a clustering analysis of microglia only to further understand their activation profile and dynamics over the disease course (Extended Data Fig. 1). We then analysed the velocity and directionality of RNA expression changes^24^ in the different cell (Fig. 1c) and microglial (Extended Data Fig. 1) clusters. Among all of the DAM clusters, DAM cluster 4 was the only one that consistently increased from control to A-EAE to C-EAE (6%, 23%, 29% of DAMs, respectively), and it showed the lowest probability of transition into other microglial clusters (that is, small RNA velocity), suggestive of a persistent (steady) state in EAE^24^. Transcriptionally, DAM cluster 4 was characterized by the increased expression of the DAM genes^22^ *Spp1* (top differentially expressed gene (DEG); Extended Data Fig. 1 and Supplementary Data 1), *Cd63*, *Cst7*, *Timp2* and *Apoe* (Fig. 1d).

**Extended Data Fig. 2:**
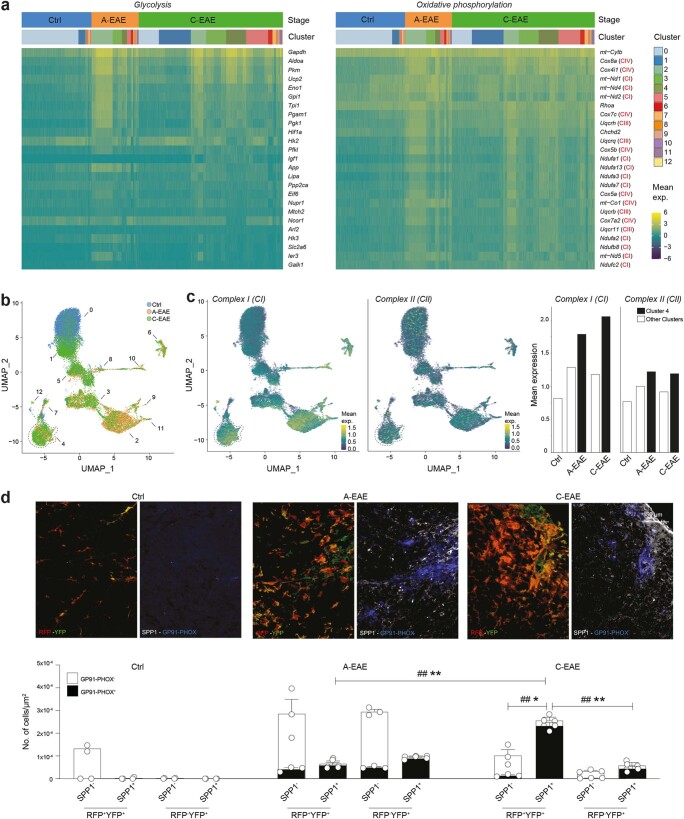
Glycolysis and oxidative phosphorylation-related genes in myeloid cells isolated from *Cx3cr1*YFP^CreERT2^:R26^tdTomato^ Ctrl and EAE mice. **a**, Heatmap of the log2-transformed counts of the top 25 differentially expressed genes (rows) involved in glycolysis and oxidative phosphorylation obtained from 22,148 cells (Ctrl = 6,205; A-EAE = 3,648; C-EAE = 12,295). Red text indicates genes of mitochondrial complex I (CI), III (CIII), and IV (CIV). **b**, **c**, UMAP plots of the whole scRNAseq dataset coloured by (**b**) stage of disease and (**c**) average expression of mitochondrial CI and CII genes (quantification is shown in the bar plots). **d**, Confocal images and quantification of the number of microglia (RFP^+^ YFP^+^) and infiltrating (RFP^-^ YFP^+^) myeloid cells expressing SPP1 and the marker of oxidative stress GP91-PHOX (n = 2, 3, 3 replicates per group; mean ± SEM; relevant statistical comparisons are shown with *P < 0.05, **P < 0.01, comparing total number of cells, and ##P < 0.01, comparing total number of GP91-PHOX^+^ cells, two-way ANOVA; Fisher’s LSD). Source Data

We performed additional transcriptomic and subclustering analyses of DAM cluster 4 to identify putative mechanisms driving its persistence in EAE. We found that DAM cluster 4 was further characterized by DEGs related to Gene Ontology (GO) terms of glycolysis (such as *Gapdh* and *Aldoa*) and oxidative phosphorylation (for example, *Cox4i1* and *Ndufa1*) (Supplementary Data 1 and Extended Data Fig. 2), while subclustering analysis identified three main subclusters (Fig. 1e and Supplementary Data 2). DAM subcluster 4.1 was defined by DEGs involved in lipid (*Fabp5*) and iron metabolism (*Fth1* and *Flt1*). DAM subcluster 4.2 was defined by DEGs associated with mitochondrial CI (for example, *mt-Nd1* and *mt-Nd4*) and cytochrome *b* (*mt-Cytb*). DAM subcluster 4.3 was defined by DEGs involved in myeloid activation (*Ccl2* and *Ccl12*) and mitochondrial metabolism (*Tspo*). GO-term analysis of DAM subclusters 4.1 and 4.3 revealed enrichment in several pathways involved in myeloid activation and metabolite signalling (Fig. 1f and Supplementary Data 2). DAM subcluster 4.2 was instead characterized by pathways associated with the electron-transport chain, mitochondrial CI function (NADH to ubiquinone) and energy-coupled proton transport against electrochemical gradient.

**Extended Data Fig. 3:**
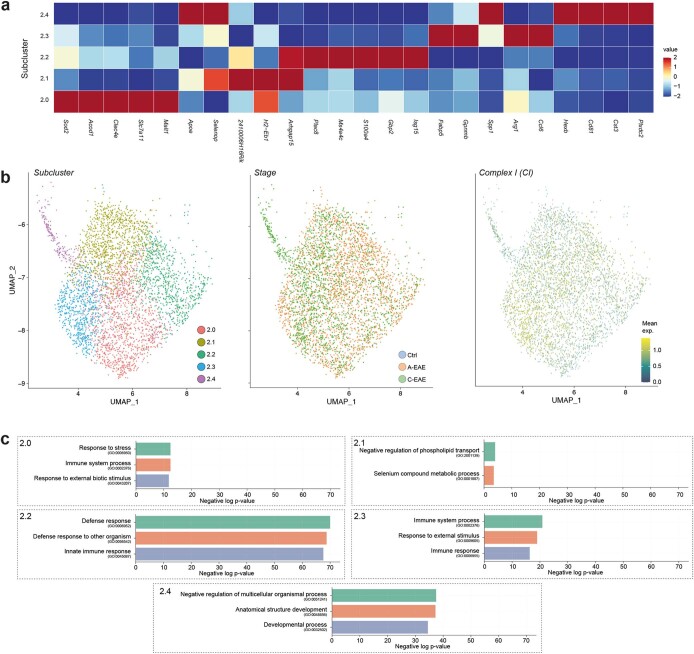
ScRNAseq unsupervised subcluster analysis of Cluster 2 isolated from *Cx3cr1*YFP^CreERT2^:R26^tdTomato^ Ctrl and EAE mice. **a**, Grouped heatmap of the average expression of the top differentially expressed genes for the 5 identified subclusters of Cluster 2. **b**, ScRNAseq UMAP plots of the subclusters of Cluster 2 coloured by (left) subcluster, (middle) stage, and (right) average expression values of mitochondrial CI genes. **c**, Top GO terms of the subclusters of Cluster 2.

Given the known functional role of CI and CII in electron transport and ROS generation in pro-inflammatory myeloid cells^12,14^, genes encoding these two mitochondrial complexes were further analysed. We found no relevant changes in the expression of genes encoding the CII subunits during the different stages of EAE in cluster 4 (Extended Data Fig. 2) or its subclusters (Fig. 1g). In A-EAE, instead, we observed an increase in genes encoding the CI subunits in DAM cluster 4 (Extended Data Fig. 2) and in cluster 2 (predominantly consisting of infiltrating myeloid cells; Extended Data Fig. 3 and Supplementary Data 2). In C-EAE, the expression of CI subunits further increased in DAM cluster 4 (Extended Data Fig. 2) and its subclusters (Fig. 1g). Pathological analysis confirmed a 7.6-fold increase in the number of SPP1^+^ DAMs expressing the NADH-ubiquinone oxidoreductase subunit of CI, NDUFS4^25^, in the spinal cord of A-EAE mice versus control mice, followed by a further 2.4-fold increase in SPP1^+^NDUFS4^+^ DAMs in C-EAE versus A-EAE mice (Fig. 1h). This finding was associated with a significant increase in SPP1^+^RFP^+^YFP^+^ microglia expressing the marker of oxidative stress GP91-PHOX^26^ in C-EAE mice (Extended Data Fig. 2).

**Extended Data Fig. 4:**
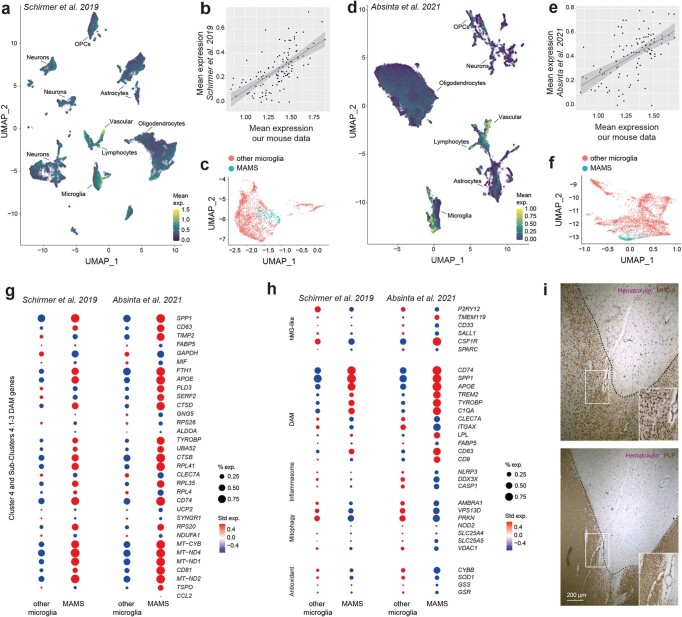
Human microglia activated in progressive MS (MAMS) display a transcriptional profile reminiscent of mouse Cluster 4 DAM. **a**, **b**, **c** The transcriptional profile of Cluster 4 DAM was consistent with a population of microglia present in MS patients, as reported in Schirmer et al. ^27^. (**a**) UMAP coloured by the average Cluster 4 DAM gene set expression. **(b**) Scatter plot comparing log average expressions for shared genes (adjusted R-squared ≈ 0.38; error bands = 0.95 confidence intervals using standard error). Regression coefficient was statistically significant. (**c**) UMAP showing the cluster of MAMS over the entire microglial population. **d**, **e**, **f**, The transcriptional profile of Cluster 4 DAM was consistent with a population of human microglia present in progressive MS patients, as reported in Absinta et al. ^28^. (**d**) UMAP coloured by the average Cluster 4 DAM gene set expression. (**e**) Scatter plot comparing log average expressions for shared genes (adjusted R-squared ≈ 0.40; error bands = 0.95 confidence intervals using standard error). Regression coefficient was statistically significant. (**f**) UMAP showing the cluster of MAMS over the entire microglial population. **g**, Bubble plot showing the expression of Cluster 4 DAM (and its sub-clusters) genes in human MAMS *vs* other microglia. **h**, Bubble plot showing hMG-like, DAM, inflammasome, positive regulation of mitophagy, and antioxidant genes in human MAMS *vs* other microglia. **i**, Staging of a representative secondary progressive MS chronic active lesion (also seen in Fig.1n) showing high MHC-II immunoreactivity specific to the lesion rim surrounding a sharply demyelinated plaque (black dotted line), as denoted by PLP staining.

To investigate the relevance of these mouse data for human disease, we reanalysed two independent publicly available single-nucleus RNA-seq datasets obtained from people with MS and control individuals post mortem^27,28^. In both datasets, we identified a cluster of human microglia activated in progressive MS (MAMS) that displayed a transcriptional profile reminiscent of the mouse DAM cluster 4 identified in our single-cell analysis. Compared with other microglia, MAMS were characterized by the high expression of DAM (for example, *SPP1* and *APOE*) and CI (such as *MT-ND1, MT-ND4*) genes, but low levels of homeostatic (for example, *P2RY12* and *SALL1)* and antioxidant genes (for example, *CYBB* and *SOD1*; Fig. 1i,j, Extended Data Fig. 4 and Supplementary Data 2). In both datasets, MAMS were almost absent in controls (Fig. 1k,l), while most MAMS were found either in chronically active (smouldering) lesions (CALs; 62% of MAMS; Fig. 1k) or at the edge of CALs (86% of MAMS; Fig. 1l) in people with MS, in whom they constituted 13% of all microglia. Pathological analysis of the brains of people with progressive MS (Extended Data Fig. 4) confirmed the presence of SPP1^+^NDUFS4^+^MHC-II^+^ myeloid cells at the CAL edge (Fig. 1m).

Thus, our research identified a cluster of persistently activated DAMs with high expression of CI genes and proteins that persists during C-EAE in mice and is found almost exclusively at the edge of CALs in people with progressive MS.

**Fig. 2:**
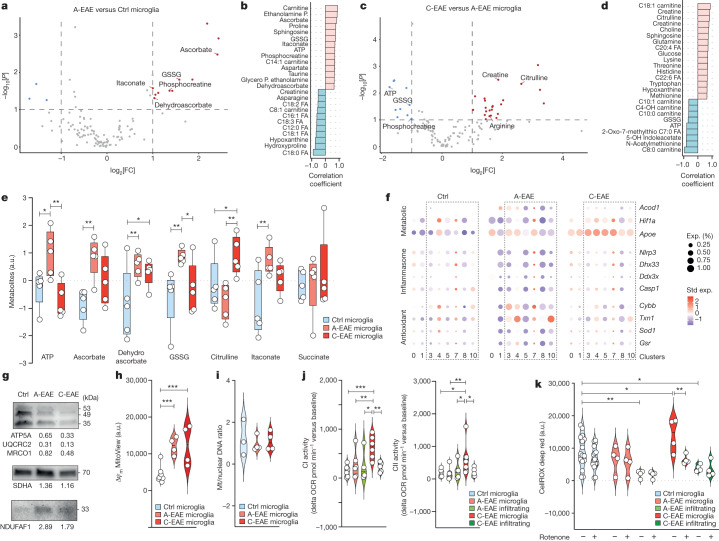
Ex vivo analysis of myeloid cells shows changes in energy metabolism, oxidative stress and mitochondrial function in EAE. **a**, The metabolites significantly altered in A-EAE versus control microglia. *n* = 5 replicates per group. Statistical analysis was performed using unpaired two-tailed *t*-tests. **b**, Corresponding correlation analysis of metabolites indicative of A-EAE versus control microglia. FA, fatty acids; P., phosphate. **c**, The metabolites significantly altered in C-EAE versus A-EAE microglia. *n* = 5 replicates per group. Statistical analysis was performed using unpaired two-tailed *t*-tests. **d**, Corresponding correlation analysis of metabolites indicative of C-EAE versus A-EAE microglia. GSSG, glutathione disulfide. **e**, Selected relevant metabolites. a.u., arbitrary units. *n* = 5 replicates per group. Statistical analysis was performed using one-way ANOVA with Fisher’s LSD test. **f**, Genes from our scRNA-seq dataset (Fig. 1a) that are involved in itaconate synthesis (*Acod1*), glycolytic switch (*Hif1a*), DAM phenotype (*Apoe*), inflammasome (*Nlrp3*, *Ddx3x*, *Dhx33*, *Casp1*), antioxidant response (*Cybb*, *Txn1*, *Sod1*) and glutathione (*Gsr*) in the microglial clusters isolated from control, A-EAE and C-EAE mice. The dotted boxes highlight DAMs. **g**–**i**, The levels of mitochondrial proteins (**g**; representative western blot, values are expressed as fold induction over the control), mitochondrial membrane potential (**h**; Δ*ψ*_m_; from left to right, *n* = 10, 4 and 4 replicates per group) and mitochondrial biogenesis (**i**; mitochondrial/nuclear DNA ratio; from left to right, *n* = 3, 4 and 4 replicates per group). Statistical analysis was performed using one-way ANOVA with Fisher’s LSD test. **j**, Mitochondrial CI and CII activity in ex vivo FACS-isolated microglia and infiltrating myeloid cells. OCR, oxygen consumption rate. From left to right, *n* = 6, 6, 4, 6 and 5 replicates per group. Statistical analysis was performed using one-way ANOVA with Fisher’s LSD test. **k**, Quantification of fluorescence intensity of the CellROX probe signal using FACS in isolated microglia and infiltrating myeloid cells treated with rotenone. From left to right, *n* = 16, 16, 4, 4, 4, 4, 4, 4, 4 and 4 replicates per group. Statistical analysis was performed using one-way ANOVA with Fisher’s LSD test. The box plots in **e** show the median (centre line), quartiles (box limits), minimum–maximum values (whiskers). The violin plots in **h**–**k** show the median and quartiles. *P < 0.05, **P< 0.01, ***P < 0.001. Source Data

**Extended Data Fig. 5:**
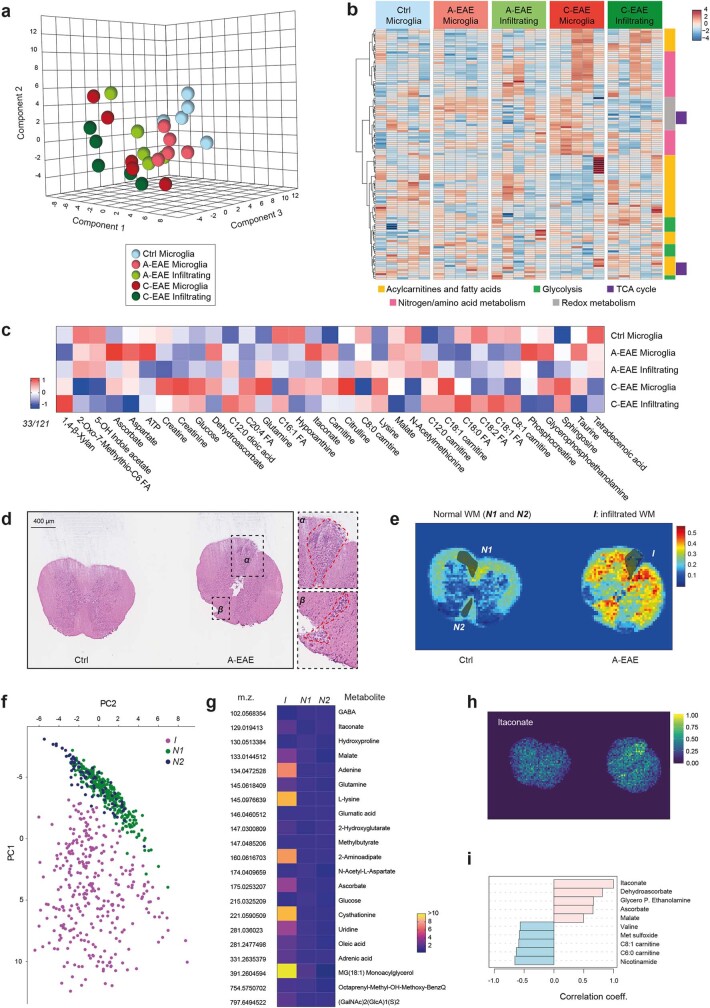
LC-MS analysis of ex vivo isolated microglia and infiltrating myeloid cells and in situ metabolomics. **a**, Partial least squares discriminant analysis 3D plot of the intracellular metabolome of microglia and infiltrating myeloid cells (n = 5 replicates per group). **b**, Heatmap depicting the metabolites (rows) identified via untargeted LC-MS analysis in each biological sample. **c**, Heatmap showing the 33/121 metabolites found to be significantly differentially regulated when comparing microglia and infiltrating myeloid cells at the different stages of EAE disease (n = 5 replicates per group; P ≤ 0.05, one-way ANOVA). Scale bars are normalized arbitrary units (A.U.). **d**, Representative haematoxylin and eosin staining (out of 6 spinal cord sections) of Ctrl and A-EAE mice used for LD-REIMS studies. Magnification of infiltrates in α and β is shown on the right. **e**, Corresponding colorimetric map based on the multivariate analysis of the spatial metabolome of spinal cord sections, as in (**d**). The infiltrated (*I*) white matter-WM (defined on haematoxylin and eosin staining) was compared with two areas of normal (*N1* and *N2)* WM. Data in the maps are shown as normalized abundances of the (tissue) spectral component extracted from the multivariate analysis. **f**, PCA plot of the metabolome of *I*, *N1*, and *N2* areas (n = 3 replicates per group). **g**, Heatmap showing the metabolites significantly changed between *I*, *N1*, and *N2* areas. Data are shown as fold change over the average values of *N1* and *N*2. **h**, Representative colorimetric maps showing the intensity of the itaconate signal in spinal cord sections as in (**d**). Data in the maps are shown as normalized abundances. **i**, Correlation analysis of itaconate with other metabolites (top 5 up/down) from the whole LC-MS dataset. P.: phosphate.

To gain further insights into the metabolic features of microglia and infiltrating myeloid cells that sustain chronic CNS inflammation, we next performed an LC–MS analysis of the intracellular metabolome of ex vivo isolated myeloid cells. We found a clear separation based on a partial least squares discriminant analysis and a differential abundance of intracellular metabolites based on the cell type and stage of EAE (Extended Data Fig. 5 and Supplementary Data 3). A-EAE microglia had increased intracellular levels of itaconate, phosphocreatine (an ATP buffer)^29^, ascorbate and dehydroascorbate (a ROS scavenger and its oxidized product), as well as glutathione disulfide (which arises from antioxidant reactions)^30^ compared with control microglia (Fig. 2a,b and Supplementary Data 3). Laser desorption-rapid evaporative ionization mass spectrometry (LD-REIMS) analysis of spinal cord sections confirmed the higher abundance of itaconate and ascorbate within white-matter inflammatory infiltrates in situ (Extended Data Fig. 5 and Supplementary Data 4). Analysis of the entire LC–MS dataset showed a direct correlation between itaconate levels and ascorbate, as well as dehydroascorbate (Extended Data Fig. 5). C-EAE microglia had instead lower itaconate and significantly lower amounts of glutathione disulfide, phosphocreatine and ATP compared with A-EAE microglia (Fig. 2c,d), which was coupled with significantly increased intracellular levels of creatine and l-citrulline (Fig. 2c–e and Supplementary Data 3).

To correlate these metabolic changes with the expression of relevant genes from our scRNA-seq dataset, we focused on the clusters of microglia isolated from control and EAE mice. The expression of *Hif1a*, which is involved in the switch to aerobic glycolysis in myeloid cells^31^, and the DAM marker *Apoe* steadily increased in DAM cluster 4 in A-EAE and C-EAE (Fig. 2f). Conversely, the expression of aconitate decarboxylase 1 (*Acod1*), which encodes the enzyme that synthesizes itaconate^16^, was increased in DAM cluster 4 in A-EAE but later decreased in C-EAE. Given the dynamic changes of intracellular phosphocreatine and antioxidants, we next focused on genes related to the inflammasome^32^ and the response to ROS. We found that the expression of genes involved in the NLRP3 inflammasome complex (such as *Nlrp3* and *Dhx33*), the antioxidant response (for example, *Cybb*, *Txn1* and *Sod1*) and glutathione synthesis/reduction (*Gsr*)^26,33^ was increased in DAM cluster 4 during A-EAE, but later decreased in C-EAE (Fig. 2f). Most of these findings are suggestive of elevated NO and ROS generation in C-EAE microglia^34^.

**Extended Data Fig. 6:**
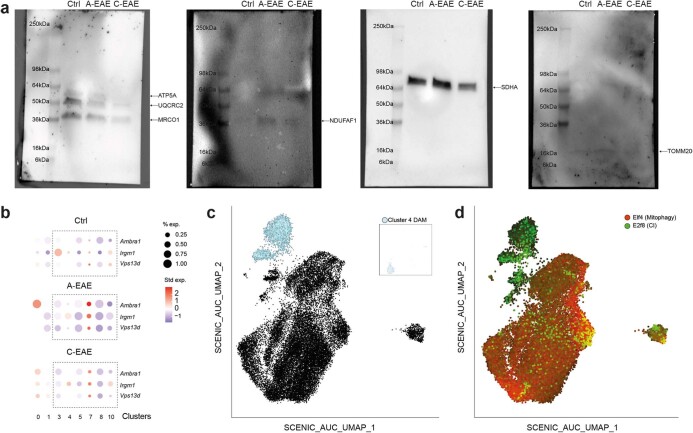
Western blot for mitochondrial proteins, mitophagy genes, and inference of gene regulatory networks (GRNs) using SCENIC. **a**, Uncropped and unprocessed scans of the Western blot reported in Fig. 2g (scans are overlayed on the colorimetric pictures to showcase molecular weight markers). **b**, Bubble plot showing genes positively regulating mitophagy in microglial clusters isolated ex vivo from EAE mice. **c**, AUC UMAP created on the *Cx3cr1*YFP^CreERT2^:R26^tdTomato^ scRNAseq dataset; light blue cells underline the link to the original Cluster 4 DAM (insert). **d**, AUC UMAP showing scores for the activity of regulons Elf4 (predicted to promote mitophagy and mitobiogenesis by targeting *Nrf1*, Liu et al. ^35^) and E2f8 (which regulates CI genes by targeting *Mybl1*, as predicted via g-profiler).

We next isolated ex vivo microglia from control, A-EAE and C-EAE mice for downstream analyses of their mitochondrial function. Mitochondrial complex III, IV and V (CIII, CIV, CV) proteins were reduced in A-EAE and C-EAE, while the CII active subunit SDHA and the CI assembly factor 1 (NDUFAF1) increased in both EAE stages versus control microglia (Fig. 2g and Extended Data Fig. 6). Although we found a significant increase in mitochondrial membrane potential starting in A-EAE and persisting in C-EAE microglia (Fig. 2h), there was no evidence of increased mitochondrial biogenesis during EAE (Fig. 2i). Instead, the reanalysis of our scRNA-seq dataset showed that genes positively regulating mitophagy (for example, *Ambra1*, *Irgm1* and *Vps13d*)^35^ were reduced in C-EAE versus A-EAE microglia (Extended Data Fig. 6). Accordingly, gene regulatory networks^36^ guiding mitophagy were downregulated in DAM cluster 4, while gene regulatory networks guiding CI transcription were upregulated (Extended Data Fig. 6). To connect these metabolic and transcriptional features with the function of mitochondrial complexes, we applied ex vivo metabolic flux analysis at different stages of disease and found that C-EAE microglia had significantly higher levels of CI and CII activity compared with control and A-EAE microglia (Fig. 2j).

On the basis of these integrated data, we propose that, after transitioning from the A-EAE to the C-EAE stage, microglia display increased CI–CII activity, reduced ATP levels and high mitochondrial membrane potential, supporting a repurposing of their mitochondria towards mtROS generation through CI, possibly via RET^12^. Consistent with this, treatment of C-EAE microglia with the CI inhibitor rotenone significantly reduced ROS production to levels observed in control mice (Fig. 2k).

**Extended Data Fig. 7:**
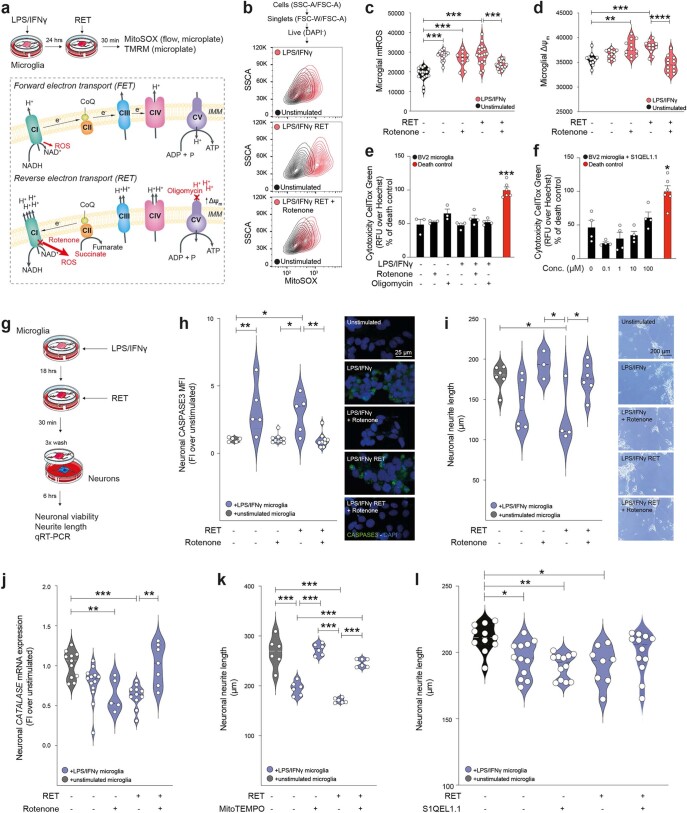
Blocking RET in microglia reduces excessive mtROS production and neurotoxicity in vitro. **a**, Experimental design for the in vitro RET studies on LPS/IFNγ stimulated microglia. IMM: inner mitochondrial membrane. **b**, Qualitative flow cytometry of mtROS production assessed via the MitoSOX probe in unstimulated and LPS/IFNγ stimulated (pro-inflammatory) BV2 microglia after RET induction (RET^+^). Rotenone is given to block CI activity. **c**, **d**, Quantification of (**c**) mtROS production (n = 18, 18, 10, 18, 18 replicates per group; one-way ANOVA; Tukey) and (**d**) mitochondrial membrane potential (Δψ_m_) in RET^+^ pro-inflammatory BV2 microglia (n = 12 replicates per group; one-way ANOVA; Tukey). **e**, Cytotoxicity assay of BV2 microglia treated with oligomycin or rotenone at the concentrations used for the in vitro experiments (n = 3, 3, 3, 4, 4, 4, 6 replicates per group; mean ± SEM; ***P < 0.001 *vs* all conditions; one-way ANOVA; Tukey). **f**, Cytotoxicity assay of BV2 microglia treated with increasing concentrations of the complex I inhibitor S1QEL1.1 in vitro (n = 4, 4, 4, 4, 4, 6 replicates per group; mean ± SEM; *P < 0.05 *vs* all conditions; one-way ANOVA; Tukey). **g**, Experimental design for the in vitro microglia-neuronal trans well co-cultures. **h**, **i**, Quantification and representative images of (**h**) CASPASE3 expression (n = 5, 5, 8, 5, 8 replicates per group; one-way ANOVA; Tukey) and (**i**) neuronal neurite length upon co-culture with RET^+^ pro-inflammatory BV2 microglia (n = 6, 6, 3, 4, 6 replicates per group; one-way ANOVA; Tukey). **j**, *CATALASE* mRNA expression in neuronal cells co-cultured with RET^+^ pro-inflammatory BV2 microglia (n = 11, 13, 5, 11, 7 replicates per group; one-way ANOVA; Tukey). **k**, Quantification of neurite length in neuronal cells pre-treated with MitoTEMPO upon co-culture with RET^+^ pro-inflammatory BV2 microglia (n = 6 replicates per group; one-way ANOVA; Tukey). **l**, Quantification of neurite length upon co-culture with LPS/IFNγ stimulated microglia (+/− RET induction +/− S1QEL1.1 inhibition) (n = 11, 12, 12, 8, 12 replicates per group; median and quartile values; one-way ANOVA; Tukey). Violin plots show median and quartiles; p values: *P < 0.05, **P < 0.01, ***P < 0.001. Source Data

To further investigate whether mitochondrial CI acts through RET to amplify the oxidative stress seen in persistent smouldering-like inflammatory CNS disease, we next applied an in vitro model of RET induction (RET^+^) to pro-inflammatory mouse microglia. RET was induced in LPS/IFNγ-stimulated microglial cells through treatment with oligomycin (which blocks mitochondrial CV and increases the mitochondrial proton motive force) in conjunction with succinate (to provide a substrate for oxidation by CII and further sustain a high mitochondrial proton motive force) (Extended Data Fig. 7). We found that the production of mtROS and increased mitochondrial membrane potential of RET^+^ pro-inflammatory microglia were both prevented by rotenone treatment without causing significant cytotoxicity (Extended Data Fig. 7). These data suggest that CI functions as a hub for mtROS production in pro-inflammatory microglia during RET in vitro.

To test the pathogenic role of RET^+^ pro-inflammatory rodent microglia, we next co-cultured them with SH-SY5Y neuronal cells using a transwell co-culture system that avoids cell-to-cell contacts (Extended Data Fig. 7). Under our conditions, we did not observe a significant change in cell death or neurite length of SH-SY5Y cells that were co-cultured with pro-inflammatory microglia without RET induction (RET^−^) (Extended Data Fig. 7). Instead, co-cultures with RET^+^ pro-inflammatory microglia were characterized by a significant increase in CASPASE3 expression, a decrease in neurite length and a downregulation of *CAT* (catalase) mRNA levels in SH-SY5Y cells, the latter being supportive of ROS-mediated damage through phosphatidylinositol 3-kinase–AKT signalling^37^ (Extended Data Fig. 7). Accordingly, pretreating SH-SY5Y cells with the ROS scavenger mitoTEMPO^38^ completely prevented RET^+^ pro-inflammatory microglial-mediated neurite damage in co-cultures (Extended Data Fig. 7), therefore supporting the predominant role of ROS in the observed microglial neurotoxicity.

Blocking CI activity in RET^+^ pro-inflammatory microglia by treatment with rotenone prevented CASPASE3 induction, loss of neurites and *CAT* induction in co-cultured SH-SY5Y cells (Extended Data Fig. 7). Conversely, blocking CI activity with the suppressor of site IQ electron leak S1QEL1.1, which inhibits the production of superoxide/hydrogen peroxide without affecting electron movement from COQH2 to NAD^18,39^, did not significantly rescue SH-SY5Y neurite length versus RET^+^ pro-inflammatory microglia (Extended Data Fig. 7).

**Extended Data Fig. 8:**
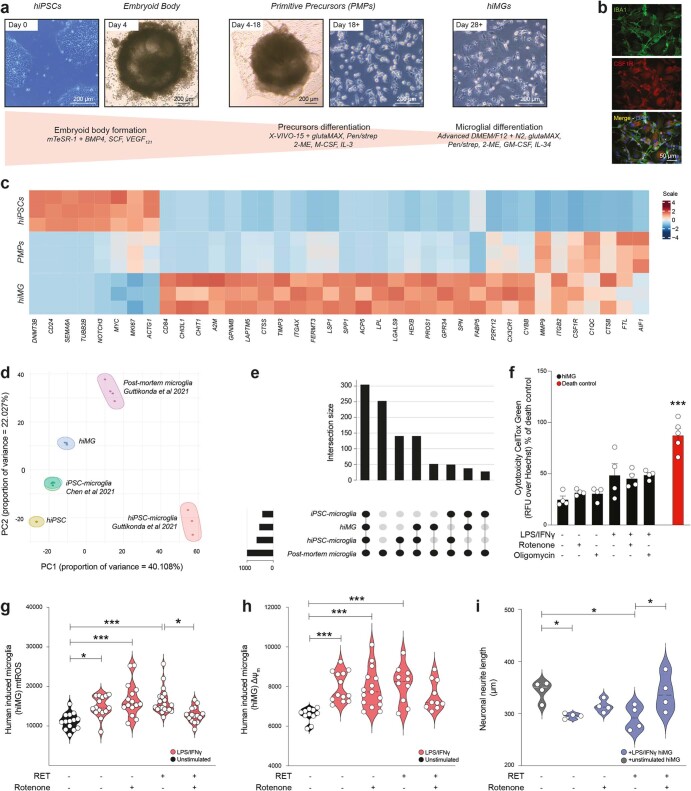
Blocking RET in human induced microglia (hiMG) reduces excessive mtROS production and neurotoxicity in vitro. **a**, Schematic diagram of the hiMG derivation protocol and relative bright field images. **b**, Representative immunofluorescence staining for IBA1 and CSF1R in hiMG. **c**, Heatmap showing the expression of top differentially regulated and other relevant genes (logfc > 1, adj pval <0.05, edgeR pipeline) in hiPSCs, intermediate primitive macrophage precursors (PMPs), and differentiated hiMG (n = 3 replicates per group). **d**, **e**, (**d**) PCA analysis and (**e**) upset plot of the top 1,000 expressed genes comparing the transcriptome of hiMG with previous published protocols used to obtain iPSC-microglia (Chen et al.^63^), hiPSC-microglia (Guttikonda et al.^62^), and *post-mortem* microglia (Guttikonda et al.^62^). PCA also shows the hiPSCs used to differentiate the hiMG following our protocol. **f**, Cytotoxicity assay of hiMG treated with oligomycin or rotenone at the concentrations used for the in vitro experiments (n = 4, 4, 4, 4, 4, 4, 5 replicates per group; mean ± SEM; ***P < 0.001 *vs* all conditions; one-way ANOVA; Tukey). **g**, **h**, Quantification of (**g**) mtROS production (n = 14, 15, 15, 20, 15 replicates per group; one-way ANOVA; Tukey) and (**h**) Δψ_m_ in RET^+^ pro-inflammatory hiMG (n = 11, 13, 15, 10, 10 replicates per group; one-way ANOVA; Tukey). **i**, Quantification of neuronal neurite length upon co-culture with RET^+^ pro-inflammatory hiMG (n = 4 replicates per group, one-way ANOVA; Bonferroni). Violin plots show median and quartiles; p values: *P < 0.05, **P < 0.01, ***P < 0.001. Source Data

We further validated the main findings obtained from mouse microglia on human induced pluripotent stem (iPS) cell-derived induced microglia (hiMGs) (Extended Data Fig. 8). RET^+^ pro-inflammatory hiMGs produced significantly more mtROS, had increased mitochondrial membrane potential and caused increased neurite toxicity in SH-SY5Y cells compared with the controls (Extended Data Fig. 8). As described for mouse microglia, these effects were all prevented by CI inhibition in hiMGs by treatment with rotenone.

Thus, blocking RET in pro-inflammatory rodent and human microglia through CI inhibition protects from excessive mtROS-associated neurotoxicity in vitro.

**Fig. 3:**
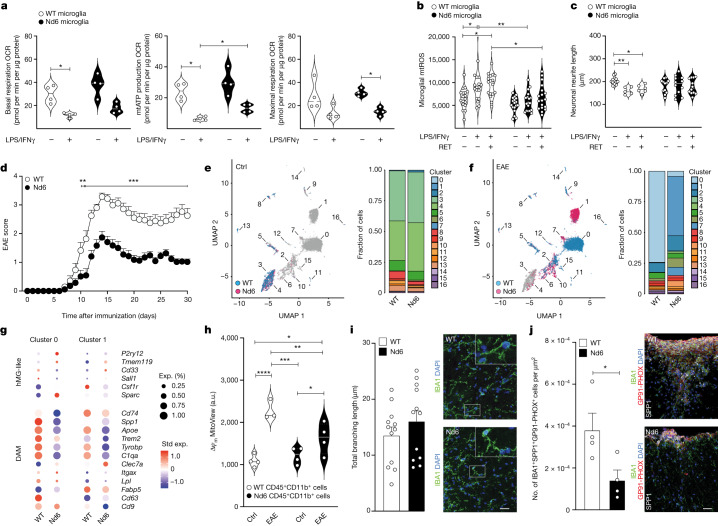
Nd6 mouse point mutation blocks RET and ameliorates EAE. **a**, Seahorse metabolic flux analysis of primary microglia derived from wild-type (WT) and Nd6 mice under basal conditions and after stimulation with LPS and IFNγ. *n* = 4 replicates per group. Statistical analysis was performed using one-way ANOVA with Tukey test. Differences in basal respiration, mitochondrial (mt) ATP production and maximal respiration are reported. **b**, Quantification of mtROS production in LPS + IFNγ-stimulated (pro-inflammatory) primary WT and Nd6 microglia after RET induction (RET^+^). From left to right, *n* = 18, 18, 17, 18, 18 and 18 replicates per group. Statistical analysis was performed using two-way ANOVA with Fisher’s LSD test. **c**, Quantification of neuronal neurite length after co-culture with RET^+^ pro-inflammatory primary WT and *Nd6* microglia. From left to right, *n* = 11, 5, 6, 12, 12, 12 replicates per group. Statistical analysis was performed using two-way ANOVA with Fisher’s LSD test. **d**, EAE scores of WT and Nd6 mice up to 30 days after immunization. *n* = 17 mice per group. Statistical analysis was performed using two-way ANOVA with Bonferroni correction. **e**,**f**, scRNA-seq UMAP plots with each cell coloured according to the genotype, obtained from 13,614 cells (7,501 (WT) and 6,113 (Nd6)). Superimposed cluster numbers and the corresponding fraction of cells are shown for control (**e**) and EAE (**f**) mice (30 days after immunization). **g**, Selected hMG-like and DAM genes in cluster 0 and 1 DAMs. **h**, The mitochondrial membrane potential (Δ*ψ*_m_) in ex vivo FACS-isolated CD45^+^CD11b^+^ cells. From left to right, *n* = 4, 3, 4 and 4 replicates per group. Statistical analysis was performed using one-way ANOVA with Fisher’s LSD test. **i**,**j** Representative images and quantifications of perilesional microglial branching (**i**; *n* = 12 replicates per group) and IBA1^+^SPP1^+^GP91-PHOX^+^ cells in WT and Nd6 EAE mice (**j**; *n* = 4 replicates per group). Statistical analysis was performed using two-tailed unpaired *t*-tests. For **i** and **j**, scale bars, 30 μm. For **d**, **i** and **j**, data are mean ± s.e.m. The violin plots in **a**–**c**, and **h** show the median and quartiles. *P < 0.05, **P < 0.01, ***P < 0.001, ****P < 0.0001. Source Data

**Extended Data Fig. 9:**
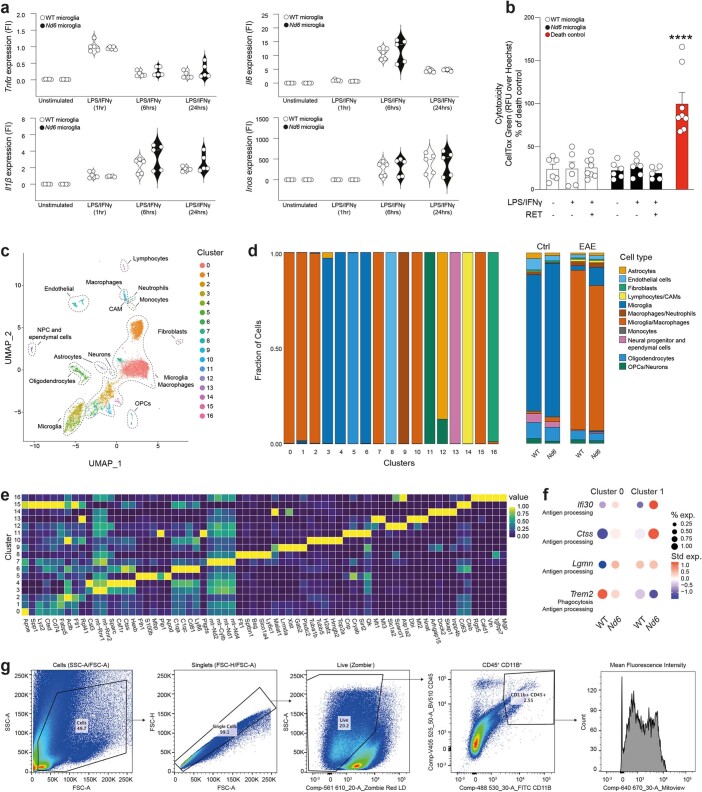
WT and *Nd6* microglia in vitro and ex vivo scRNAseq characterization of the CNS in WT and *Nd6* mice. **a**, Quantitative RT-PCR showing the levels of *Tnfα, Il1β, Il6*, and *Inos* expression in primary microglia derived from WT or *Nd6* mice (n = 5 replicates per group; median and quartile values; two-way ANOVA; Sidak). Data are normalized over WT microglia stimulated with LPS/IFNγ. **b**, Cytotoxicity assay of WT and *Nd6* primary microglia treated after LPS/IFNγ +/− RET induction (n = 6, 6, 8, 6, 6, 5, 8 replicates per group; mean ± SEM; ****P < 0.0001 *vs* all conditions; one-way ANOVA; Tukey). **c**, UMAP plot coloured by cluster of cells isolated from WT and *Nd6* (healthy Ctrl and EAE at 30 dpi) mice. **d**, (left) relative proportion of cell types within the 17 clusters and (right) corresponding proportion of cell types in WT *vs Nd6* healthy Ctrl and EAE mice. **e**, Heatmap of the average log2-transformed counts of top differentially expressed genes across the 17 clusters. **f**, Bubble plot summarizing the expression level of significantly DEGs [belonging to the phagocytosis (GO:0006909), antigen processing and presentation (GO:0019882) and growth factor activity (GO:0008083) pathways] in Cluster 0 and 1 DAM comparing WT *vs Nd6* EAE mice. **g**, Gating strategy to measure mitochondrial membrane potential using the MitoView633 probe in the CD45^+^Cd11b^+^ myeloid cells from WT and *Nd6* mice. Source Data

To further establish the role of CI and RET in microglial polarization and function, we next isolated primary microglia from Nd6 mice. These mice carry a point mutation in the mitochondrial CI gene *Nd6* that blocks RET while preserving normal forward electron transport^40^. Microglial stimulation with LPS and IFNγ induced superimposable effects in the expression of genes coding for major pro-inflammatory cytokines in both wild-type (WT) and Nd6 microglia (Extended Data Fig. 9), while stimulated Nd6 microglia displayed significantly higher mitochondrial ATP production (Fig. 3a). Nd6 microglia did not increase mtROS production (Fig. 3b) or cause significant neurite toxicity in co-culture with of SH-SY5Y cells (Fig. 3c) under in vitro conditions that forcing RET in microglia (as in Extended Data Fig. 7).

To verify the relevance of these findings in a disease model in vivo, we induced MOG_35–55_ EAE in Nd6 and WT mice. Nd6 mice developed a significantly milder EAE throughout the entire disease course compared with WT mice (Fig. 3d). Ex vivo scRNA-seq analysis of the entire CNS of WT and Nd6 mice identified 17 cell clusters in non-immunized control and EAE mice (Fig. 3e,f and Extended Data Fig. 9). In non-immunized control mice, we found a 1.3-fold increase in cluster 4 hMG-like cells in Nd6 versus WT mice (Fig. 3e and Supplementary Data 5). In EAE, Nd6 mice displayed a 14.0-fold and a 4.0-fold increase in hMG-like clusters 3 and 4, respectively (Fig. 3f). Furthermore, Nd6 EAE mice showed substantial changes in DAM phenotypes (Supplementary Data 5), the most notable ones being a 23.2-fold reduction of cluster 0 DAM (characterized by the expression of *Apoe* and *Spp1*) and a 101-fold increase in cluster 1 DAM (characterized by *Fabp5* expression). When comparing the expression of known hMG-like and DAM genes in these two clusters, a reduction in the expression of *Trem2* and *Apoe* was found in Nd6 EAE mice (Fig. 3g). The same cluster 0 and cluster 1 DAMs also showed a significant increase in genes associated with mitochondrial ATP-synthesis-coupled electron transport (such as *mt-Nd2* and *mt-Co1*) (Supplementary Data 5), an increased expression of lysosomal genes involved in antigen processing and presentation (for example, *Ifi30* and *Ctss*), but no significant DEGs related to growth-factor activity (Extended Data Fig. 9).

We next isolated CD45^+^Cd11b^+^ myeloid cells from non-immunized control and EAE mice to assess their mitochondrial membrane potential ex vivo (Fig. 3h and Extended Data Fig. 9). In non-immunized control mice, we found no difference in mitochondrial membrane potential between the Nd6 and WT groups. In EAE, myeloid cells isolated from Nd6 mice had a significantly lower mitochondrial membrane potential compared with WT mice. Pathologically, despite not observing significant morphological differences in perilesional microglia^41^ in Nd6 and WT EAE mice (Fig. 3i), we found a significant decrease in SPP1^+^IBA1^+^ cells expressing GP91-PHOX^26^ in the spinal cords of Nd6 EAE mice (Fig. 3j).

Thus, the lack of RET in Nd6 mice actively regulates microglial responses to neuroinflammation, which results in a reduction in oxidative stress in vivo.

**Extended Data Fig. 10:**
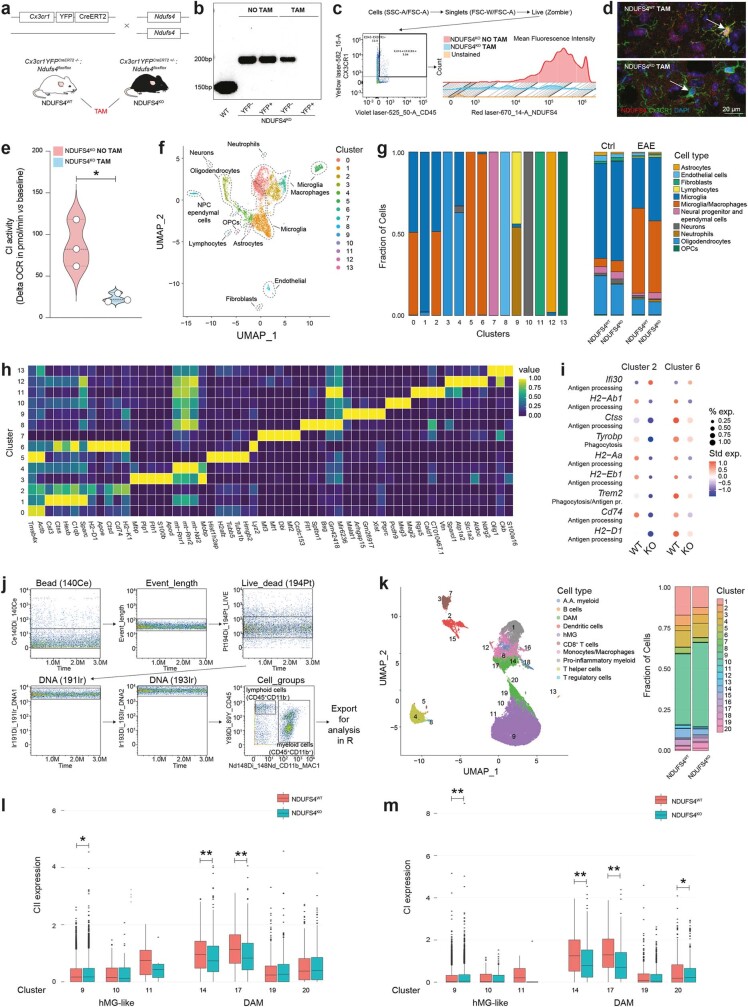
Characterization of the *Cx3cr1*YFP^CreERT2^*:Ndufs4*^flox/flox^ mice. **a**, Experimental paradigm for the in vivo and ex vivo experiments on *Cx3cr1*YFP^CreERT2−/−^*:Ndufs4*^flox/flox^ (NDUFS4^WT^) and *Cx3cr1YFP*^CreERT2+/−^*:Nduf4*^flox/flox^ (NDUFS4^KO^) mice. A 5-day course of intraperitoneal tamoxifen (TAM) injections is used to induce transgene activation. **b**, Genomic PCR for *Ndufs4* in CX3CR1^+^ cells isolated via FACS from the CNS of healthy NDUFS4^KO^ mice 2 days after TAM or saline treatment (NO TAM). **c**, Representative flow cytometry showing the NDUFS4 protein expression in CX3CR1^+^ cells isolated from the CNS of healthy NDUFS4^KO^ mice 30 days after TAM or NO TAM. **d**, Representative confocal images of NDUFS4 protein expression in CX3CR1^+^ cells of NDUFS4^WT^ and NDUFS4^KO^ healthy mice 30 days after TAM. **e**, CI activity in NDUFS4^KO^ EAE mice. TAM treated NDUFS4^KO^ EAE mice showed a 70% reduction of CI activity in CX3CR1^+^ cells at C-EAE compared to NO TAM (n = 3 replicates per group; *P < 0.05; unpaired t-test, two-tailed). **f**, UMAP plot coloured by cluster of cells isolated from NDUFS4^WT^ and NDUFS4^KO^ (healthy Ctrl and EAE at 50 dpi) mice. **g**, (left) relative proportion of cell types within the 14 clusters and (right) corresponding proportion of cell types in NDUFS4^WT^ *vs* NDUFS4^KO^ healthy Ctrl and EAE mice. **h**, Heatmap of the average log2-transformed counts of top differentially expressed genes across the 14 clusters. **i**, Bubble plot summarizing the expression level of significantly DEGs [belonging to the phagocytosis (GO:0006909), antigen processing and presentation (GO:0019882) and growth factor activity (GO:0008083) pathways] in Cluster 2 and 6 DAM comparing NDUFS4^WT^ *vs* NDUFS4^KO^ mice EAE mice. **j**, Gating strategy for the CyTOF analyses. **k**, UMAP plot from the CyTOF data coloured by cell type found in NDUFS4^WT^ and NDUFS4^KO^ EAE mice at 50 dpi. Numbers of clusters are shown in superimposition. A.A.: alternatively activated. The fraction of cell clusters is shown on the right. **l**, **m**, Expression of (**l**) CII and (**m**) CI in the clusters identified as hMG-like cells and DAM (median and quartiles from 51,177 cells; *P < 0.05, **P < 0.01; unpaired t-test, two-tailed). Source Data

To investigate the possibility of therapeutically targeting CI in myeloid cells only during the transition between A-EAE and C-EAE, we next generated tamoxifen-inducible transgenic mice that allow for the timed knockout of *Ndufs4*^42^ in CX3CR1^+^ cells in vivo (Extended Data Fig. 10). We induced MOG_35–55_ EAE in *Cx3cr1-YFP*^*creERT2*^*Ndufs4*^*flox/flox*^ mice and administered tamoxifen 1 week after the onset of the EAE clinical signs to obtain *Ndufs4*-KO mice.

**Fig. 4:**
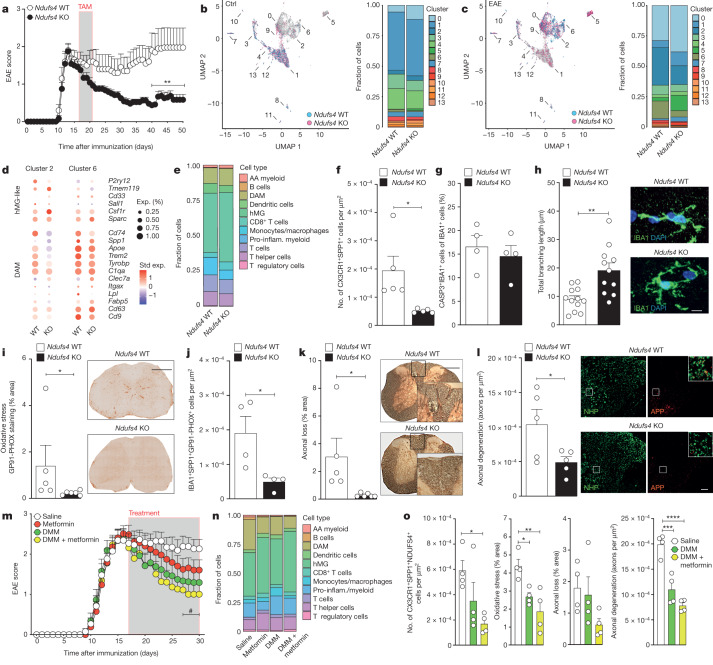
Targeting RET in vivo ameliorates EAE and reduces secondary axonal damage. **a**, EAE scores of *Ndufs4-*WT and *Ndufs4*-KO mice. *n* = 10 (WT) and 12 (KO) mice per group. Statistical analysis was performed using two-way ANOVA with Bonferroni correction. **b**,**c**, scRNA-seq UMAP plots with each cell coloured according to the genotype, obtained from 10,666 cells (4,180 (*Ndufs4* WT) and 6,486 (*Ndufs4* KO)). Superimposed cluster numbers and the corresponding fraction of cells are shown for control (**b**) and EAE (**c**) mice (50 days after immunization). **d**, Selected hMG-like and DAM genes in cluster 2 and 6 DAMs. **e**, Suspension mass cytometry (CyTOF) analysis of immune cell types at 50 days after immunization obtained from 51,177 cells (23,467 (*Ndufs4* WT); 27,710 (*Ndufs4* KO)). AA, alternatively activated; pro-inflam., pro-inflammatory. **f**,**g**, Quantification of CX3CR1^+^SPP1^+^ (**f**; *n* = 5 replicates per group) and CASPASE3^+^IBA1^+^ (**g**; *n* = 4 replicates per group) cells in EAE. Statistical analysis was performed using two-tailed unpaired *t*-tests. **h**–**j**, In vivo quantification of perilesional microglial branching (**h**; *n* = 12 (WT) and 11 (KO) replicates per group; two-tailed unpaired *t*-test), GP91-PHOX expression in the EAE spinal cords (**i**; *n* = 5 (WT) and 6 (KO) replicates per group; two-tailed Mann–Whitney *U*-test) and IBA1^+^SPP1^+^GP91-PHOX^+^ cells (**j**; *n* = 4 replicates per group; two-tailed unpaired *t*-test). Scale bars, 7 μm (**h**) and 400 μm (**i**). **k**,**l**, Representative images and quantification of axonal loss (**k**; *n* = 5 (WT) and 6 (KO) replicates per group) and axonal degeneration (**l**; n = 5 replicates per group). Statistical analysis was performed using two-tailed unpaired *t*-tests. APP, amyloid precursor protein; NHP, neurofilament heavy polypeptide. Insets: merged images. Scale bars, 400 μm (**k**) and 50 μm (**l**). **m**, EAE scores of mice treated with metformin, DMM, DMM + metformin versus saline controls. *n* = 13 mice per group. Statistical analysis was performed using two-way ANOVA with Bonferroni correction; ^#^*P* < 0.05 comparing DMM + metformin versus saline. **n**, CyTOF analysis of immune cell types at 30 days after immunization obtained from 159,110 cells (32,793 (metformin), 40,864 (DMM), 44,143 (DMM + metformin) and 41,310 (saline)). **o**, Quantification of CX3CR1^+^SPP1^+^NDUFS4^+^ cells, oxidative stress, axonal loss and axonal degeneration in EAE mice. *n* = 4 replicates per group. Statistical analysis was performed using one-way ANOVA with Tukey test. For **a**, **f**–**m** and **o**, data are mean ± s.e.m. *P < 0.05, **P < 0.01, ***P < 0.001, ***P < 0.0001. Source Data

We found that *Ndufs4*-KO EAE mice had significantly lower disease severity when they reached the C-EAE stage compared with *Ndufs4*-WT EAE mice (Fig. 4a). Ex vivo scRNA-seq of the entire CNS of *Ndufs4-*WT and *Ndufs4*-KO mice revealed 14 cell clusters in non-immunized control and EAE mice (Fig. 4b,c, Extended Data Fig. 10 and Supplementary Data 6). In non-immunized control mice, *Ndufs4*-KO mice showed a slight decrease (1.1-fold) in cluster 3 oligodendrocytes and an increase in cluster 0 microglia (2.6-fold), which was characterized by genes involved in cytoskeletal organization such as β-actin (*Actb*) and thymosin β-4 (*Tmsb4x*)^43–46^ (Fig. 4b and Supplementary Data 6). In EAE, *Ndufs4*-KO mice had a 2.6-fold increase in cluster 0 microglia, an 8.1-fold increase in cluster 5 microglia (also characterized by cytoskeletal genes, such as tubulin β-5 chain (*Tubb5*)) and a 4.9-fold increase in cluster 7 neural progenitor/ependymal cells (Fig. 4c and Supplementary Data 6). In EAE, *Ndufs4-*KO mice also showed a 3.6-fold increase of cluster 1 hMG-like cells, which was coupled with a significant reduction in cluster 2 and cluster 6 DAMs expressing *Apoe* and *Cd74* (1.1- and 1.6-fold, respectively). When comparing the expression of known hMG-like and DAM genes in these two latter clusters, we found that *Ndufs4*-KO EAE mice had reduced DAM gene expression (for example, *Cd74*, *Spp1* and *Apoe*) and increased homeostatic gene expression (for example, *Csf1r* and *Sparc*) (Fig. 4d and Supplementary Data 6). Clusters 2 and 6 showed no significant DEGs related to growth factor activity, but showed a reduction in the expression of genes involved in phagocytosis and antigen processing compared with *Ndufs4*-WT EAE mice, except for *Ifi30* (Extended Data Fig. 10).

Ex vivo mass cytometry analysis of CD45^+^ spinal cord immune cells (Supplementary Data 7) confirmed a concordant 17% increase in the number of hMG-like cells (Fig. 4e) coupled with a lower CII and CI expression in DAMs isolated from *Ndufs4*-KO EAE mice (Extended Data Fig. 10). Pathologically, *Ndufs4*-KO EAE mice showed a significant reduction in CX3CR1^+^SPP1^+^ cells in the spinal cord (Fig. 4f) but no significant difference of apoptotic IBA1^+^ cells (Fig. 4g). *Ndufs4*-KO EAE mice also showed a significant increase in perilesional microglial branching (Fig. 4h), which was consistent with the differences in genes regulating cytoskeletal organization found in our scRNA-seq dataset. Finally, *Ndufs4*-KO EAE mice showed significantly diminished oxidative stress in the spinal cord (Fig. 4i), a reduction in GP91-PHOX expression in SPP1^+^IBA1^+^ cells (Fig. 4j) and significantly lower axonal loss and degeneration^47^ (Fig. 4k,l).

Thus, targeting CI activity in myeloid cells in vivo increases hMG-like cells and induces DAM changes that together prevent oxidative stress and associated neurotoxicity.

**Extended Data Fig. 11:**
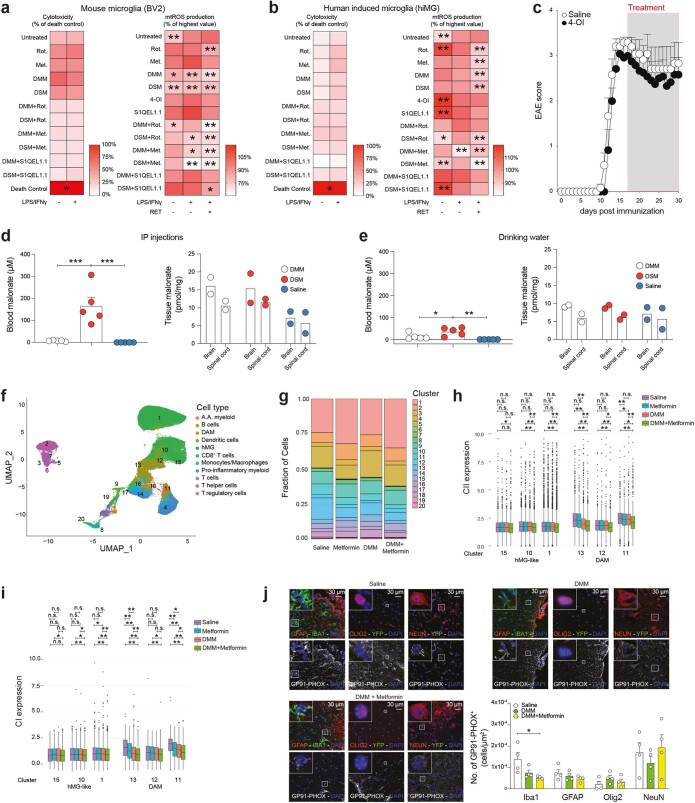
CI and CII inhibitors in vitro and in vivo. **a**, Cytotoxicity assay and mtROS production of mouse BV2 microglia treated with selected CI and/or CII inhibitors in vitro. Cytotoxicity assay data are expressed as % of death control from n = 7, 3, 3, 3, 4, 4, 4, 4, 4, 4, 4, 15 replicates per group (first column, top-bottom), n = 8, 4, 4, 4, 4, 4, 4, 4, 4, 4, 4, 15 replicates per group (second column, top-bottom); *P < 0.05 *vs* death control; one-way ANOVA; Tukey). MtROS data is normalized on RET and shown as % increase or decrease *vs* RET mtROS baseline (assessed via MitoSOX) from n = 37, 11, 14, 11, 11, 10, 11, 6, 6, 6, 3, 11, 11 replicates per group (first column, top-bottom), n = 46, 11, 14, 11, 11, 18, 17, 5, 6, 6, 3, 18, 18 replicates per group (second column, top-bottom), n = 38, 20, 16, 20, 20, 18, 18, 8, 8, 8, 4, 18, 18 replicates per group (third column, top-bottom); *P < 0.05, **P < 0.01 *vs* untreated RET^+^ pro-inflammatory microglia; unpaired t-test, two-tailed. **b**, Cytotoxicity assay and mtROS production of human induced microglia (hiMG) treated with selected CI and/or CII inhibitors in vitro. Cytotoxicity assay data are expressed as % of death control from n = 8, 4, 4, 4, 4, 4, 4, 4, 4, 4, 4, 16 replicates per group (first column, top-bottom), n = 7, 4, 3, 4, 4, 4, 4, 4, 4, 4, 4, 16 replicates per group (second column, top-bottom); *P < 0.05 *vs* death control; one-way ANOVA; Tukey). MtROS data is normalized on RET and shown as % increase or decrease *vs* RET mtROS baseline (assessed via MitoSOX) from n = 27, 10, 10, 10, 10, 6, 6, 8, 8, 8, 8, 6, 6 replicates per group (first column, top-bottom), n = 32, 26, 10, 10, 10, 6, 6, 8, 8, 8, 8, 6, 6 replicates per group (second column, top-bottom), n = 31, 26, 10, 10, 10, 6, 6, 8, 8, 8, 8, 6, 5 replicates per group (third column, top-bottom); *P < 0.05, **P < 0.01 *vs* untreated RET^+^ pro-inflammatory microglia; unpaired t-test, two-tailed. **c**, EAE scores of mice treated with 4-octyl itaconate (4-OI) *vs* saline controls (n = 6 mice per group; mean ± SEM; two-way ANOVA; Bonferroni). **d**, **e**, In vivo testing of dimethyl malonate (DMM) and disodium malonate (DSM) in EAE. (**d**) EAE mice receiving daily intraperitoneal (IP) injections of DMM (160 mg/kg), DSM (160 mg/kg), or saline at 7 days from disease onset. Malonate levels in the peripheral blood (at 30 min from injection, left; n = 5 replicates per group; mean ± SEM; one-way ANOVA; Tukey) and in the CNS (at sacrifice, right; n = 2 replicates per group; mean). ***P < 0.001. (**e**) EAE mice receiving DMM (1.5%), DSM (1.5%), or saline dissolved in their drinking water (at 7 days from disease onset). Malonate levels in the peripheral blood during treatment (daily, left; n = 5 replicates per group; mean ± SEM; one-way ANOVA; Tukey) and in the CNS (at sacrifice, right; n = 2 replicates per group; mean). *P < 0.05, **P < 0.01. **f**, UMAP plot from the CyTOF data at 30 dpi (see also Fig. 4n) coloured by cell type found in EAE mice treated with CII and CI inhibitors. Numbers of clusters are shown in superimposition. A.A.: alternatively activated. **g**, Fraction of cell clusters in EAE mice treated with metformin, DMM, DMM+metformin, *vs* saline-treated controls at 30 dpi. **h**, **i**, Expression of (**h**) CII and (**i**) CI in the CyTOF clusters identified as hMG-like cells and DAM (median and quartiles from 159,110 cells; *P < 0.01, **P < 0.0001; unpaired t-test, two-tailed). **j**, Representative confocal images and quantification of the expression of the marker of oxidative stress GP91-PHOX in microglia (IBA1^+^), astrocytes (GFAP^+^), oligodendrocytes (OLIG2^+^), and neurons (NEUN^+^) in the spinal cord of treated EAE mice (n = 4 replicates per group; mean ± SEM; *P < 0.05; one-way ANOVA; Tukey). Abbreviations: rotenone (rot.), metformin (met.), 4-octyl itaconate (4-OI), dimethyl malonate (DMM), disodium malonate (DSM). Source Data

We next performed in vitro drug testing in mouse and human microglia to select inhibitors of CI and CII activity for in vivo testing (Extended Data Fig. 11). The CI inhibitors rotenone and metformin^48^, as well as the CII inhibitors dimethyl malonate (DMM) and disodium malonate, were the most effective in reducing mtROS production in vitro. This effect was further enhanced by the combination of selected CI and CII inhibitors.

In vivo, daily intraperitoneal injections of 4-octyl itaconate^49^ (a weak CII inhibitor^50^) (Extended Data Fig. 11) or DMM only (Fig. 4m) did not ameliorate MOG_35–55_ EAE in mice. Instead, the combination of DMM + metformin led to the most significant therapeutic effect on EAE at 30 days after immunization (Fig. 4m). Ex vivo mass cytometry analysis of CD45^+^ spinal cord leukocytes revealed a marked effect of DMM + metformin on the frequency of hMG-like (1.4 and 1.3-fold increase, versus saline and DMM, respectively) and DAM (2.5 and 1.6-fold decrease, versus saline and DMM, respectively) (Fig. 4n), coupled with a significant reduction in CII and CI expression in DAM clusters (Extended Data Fig. 11). Pathologically, treatment with DMM + metformin led to a significant decrease in CX3CR1^+^SPP1^+^NDUFS4^+^ cells, which was coupled with reduced oxidative stress in the spinal cord (Fig. 4o), a reduction in GP91-PHOX^+^ expression in IBA1^+^ cells only (Extended Data Fig. 11) and a significant protection from axonal loss and degeneration (Fig. 4o).

Although the role of mitochondria in MS is extensively described in neurons^8–11^, our study identifies a key mitochondrial mechanism that contributes to the perpetuation of CNS inflammation by sustaining microglial activation and neurotoxic damage. We show that, as CNS inflammation becomes chronic, microglia display lower phosphocreatine and ATP levels that are coupled with transcriptomic changes indicative of a lower antioxidant and inflammasome response. This aligns with previous data suggesting a link between phosphocreatine depletion, low ATP levels and decreased NLRP3 inflammasome activation^32^. However, after transitioning from the A-EAE to the C-EAE stage, microglia also show reduced itaconate levels (a known CII inhibitor)^50,51^, higher *Hif1a* transcription and a progressive alteration of their mitochondrial function.

Compelling evidence exists that succinate oxidation, coupled with elevated mitochondrial potential, is instrumental for the generation of RET^12^ in vitro. In the context of acute ischaemia–reperfusion injury in vivo, succinate accumulates during ischaemia (when the electron-transport chain is obstructed due to the absence of oxygen) and after reperfusion the oxidation of the succinate pool drives RET^17^. Under our chronic experimental conditions in vivo, we did not anticipate a long-term build-up of succinate. Rather, we propose that heightened succinate oxidation, supported by increased CII activity, in conditions characterized by a high mitochondrial membrane potential and diminished ATP synthesis, propels RET through CI in microglia. Accordingly, we find that exposing pro-inflammatory mouse and human microglia in vitro to conditions that boost RET leads to increased oxidative stress and paracrine neurotoxicity that is dependent on CI activity.

Mechanistically, we show that this process is prevented in vitro by blocking CI with small molecules or by using Nd6 microglia that have no RET. In an animal disease model in vivo, interfering with the function of the subunits of mitochondrial CI promotes the acquisition of a protective hMG-like phenotype. Moreover, it shifts the transcriptional profile of diverse DAM populations, which together result in the prevention of oxidative stress and neurotoxicity that lead to a protection from irreversible disabilities in mice. Cell-lineage-specific NDUFS4 conditional loss-of-function experiments and in vivo small-molecule treatments reinforce the rationale for innovative therapeutic strategies designed to reduce mitochondrial CI activity within myeloid cells. This offers a promising route for targeting and resolving long-term CNS inflammation.

In conclusion, our study identifies a mechanism that sustains microglial activation that is therapeutically actionable. Our findings are also extremely timely and of potential clinical relevance given the ongoing clinical trials (for example, NCT05131828, NCT04121468, NCT05893225) testing the therapeutic effects of the CI inhibitor metformin in people with both relapsing remitting and progressive MS.

## Methods

### Mice and EAE

The *Cx3cr1-YFP*^*creERT2*^*R26*^*tdTomato*^ fate mapping mouse was generated by crossing B6.129P2(Cg)-Cx3cr1tm2.1(cre/ERT2)Litt/WganJ^52^ with B6.Cg-Gt(ROSA)26Sortm9(CAG-tdTomato)Hze/J)^53^, as previously described^19,20^. ND6-P25L mice mice (referred to here as Nd6 mice) were generated as described previously^40^ and were obtained from D. Wallace. The *Cx3cr1-YFP*^*creERT2*^*Ndufs4*^*flox/flox*^ mice were generated by crossing B6.129P2(Cg)-Cx3cr1tm2.1(cre/ERT2)Litt/WganJ^52^ with mice with conditional alleles of the *Ndufs4* gene (exon 2 flanked by *loxP* sites)^42^. Wild-type C57BL/6 female mice were purchased from Charles River. EAE induction was performed by immunization with MOG_35–55_, as previously described^13^.

To induce RFP expression in *Cx3cr1-YFP*^*creERT2*^*R26*^*tdTomato*^ mice, tamoxifen (TAM) (Sigma-Aldrich) was diluted in corn oil (Sigma-Aldrich) at a concentration of 25 mg ml^−1^ and administered daily through intraperitoneal (i.p.) injections for 5 days at 0.125 mg TAM per g body weight (2.5 mg per 20 g mouse). After a washout period of 28 days to allow peripheral myeloid cells to be replaced de novo (and therefore lose RFP expression)^54^, EAE was induced.

To knockout *Ndufs4* expression in CX3CR1^+^ cells, TAM was diluted in corn oil and administered through daily i.p. injections for 5 days (as above) starting 1 week after EAE onset to *Cx3cr1YFP*^*creERT2−/−*^*Ndufs4*^*flox/flox*^ (*Ndufs4* WT) and *Cx3cr1-YFP*^*creERT2+/−*^*Ndufs4*^*flox/flox*^ (*Ndufs4* KO) mice. EAE induction was performed on female mice aged 8–20 weeks. Healthy controls included both female and male mice. For tamoxifen and small molecule treatments of EAE mice, mice were randomly assigned to each treatment group on the first day of treatment (one week after onset), so that the mean baseline EAE score of each group was not statistically different. In all other occasions, mice and samples were randomly selected by blind assessors. Investigators assessing in vivo (for example, behavioural), ex vivo (for example, pathological), and in vitro (cell cultures) outcomes were blinded to group allocation. Data from published^13^ and preliminary work has been used to obtain estimates of standard deviation (s.d.) and population distribution. Sample size calculations were carried out for an effect size of 0.5, 80% power, 5% level of significance, and for highest s.d. of the outcomes assessed.

Body weight and EAE clinical score were recorded daily. At 7–16 days after immunization, mice developed the first clinical signs of disease (EAE onset), and at 3 days after disease onset, they reached the acute phase of disease (A-EAE). EAE scoring (0, healthy; 1, limp tail; 2, ataxia and/or paresis of hindlimbs; 3, paralysis of hindlimbs and/or paresis of forelimbs; 4, quadriplegia; 5, found dead) was adapted to include 0.25 increments in case of mice exceeding the conventional score, and a score of 4.5 was given to mice that reached the severity limit of our licence and had to be culled humanely. During the experiments, mice were housed in ventilated cages, softly lit and subject to a light dark cycle with a relative humidity of 45–65% and at a temperature range of 20–24 °C.

### scRNA-seq

For *Cx3cr1-YFP*^*creERT2*^*R26*^*tdTomato*^ scRNA-seq experiments, mice were deeply anaesthetized with an i.p. injection of ketamine (10 mg ml^−1^, Boehringer Ingelheim) and xylazine (1.17 mg ml^−1^, Bayer) and perfused with ice-cold artificial CSF (aCSF). After perfusion, spinal cords were extracted from the spinal columns using a 5 ml syringe filled with ice-cold aCSF. Spinal cords were mechanically dissociated in a glass Dounce tissue homogenizer with 6 ml of homogenization buffer (aCSF plus 10 mM HEPES (Sigma-Aldrich), 1% bovine serum albumin (BSA) (Sigma-Aldrich), 1 mM EDTA (Thermo Fisher Scientific), 10 mg ml^−1^ of DNase (3000U, Roche) and 40 U μl^−1^ of RNase inhibitor (Invitrogen)). After tissue dissociation, the suspension was filtered through a pre-wet 40 μM strainer and the homogenizer rinsed with 2 ml of homogenization buffer. The samples were then transferred to 15 ml Falcon tubes and 2.7 ml of 90% Percoll (GE Healthcare) in 10× PBS (Thermo Fisher Scientific) was added to each sample to remove myelin and debris. The 15 ml Falcon tubes were then inverted ten times gently and the samples were centrifuged at 800*g* for 20 min at 4 °C with a brake speed of 0. Myelin debris visibly layered at the surface was carefully removed with a pipette. Ice-cold buffer (95% autoMACS rinsing solution (Miltenyi Biotec) and 5% MACS BSA (Miltenyi Biotec)) was added to the samples (to fill the 15 ml tubes) and the samples were centrifuged at 800*g* for 5 min at 4 °C to remove the remaining Percoll. The pellets were resuspended in ice-cold aCSF (1 ml + 7 ml) and then further centrifuged at 800*g* for 5 min at 4 °C. Pelleted cells were resuspended in 200 μl of FACS buffer (Cell Staining Buffer (BioLegend) plus 10 mM HEPES, 1% BSA, 1 mM EDTA, 10 mg ml^−1^ of DNase and 40 U μl^−1^ of RNase inhibitor) and 7-AAD live/dead stain (Thermo Fisher Scientific) added at a concentration of 1:50.

The samples were sorted using a BD FACS Aria III cell sorter set to 3-way purity with a 100 μm nozzle at 20 psi using the FACS gating strategy summarized in Extended Data Fig. 1b. In brief, live cells gates were set based on the unstained WT sample, 7-AAD-stained WT sample treated with DMSO (positive control for cell death) and non-TAM-treated *Cx3cr1-YFP*^*creERT2*^*R26*^*tdTomato*^ samples (to control for bleed-through). FACS-isolated cells were sequenced at a single-cell resolution using the v3 10x Genomics Chromium Single Cell 3′ Solution. Up to 18,000 cells per sample were loaded into each well and the resultant libraries were sequenced on the NovaSeq 6000 (Illumina) system to depth of at least 50,000 reads per cell as calculated by Cell Ranger.

For *Nd6* and *Ndufs4* scRNA-seq experiments, mice were perfused with actinomycin D (5 mg ml^−1^, Sigma-Aldrich) and triptolide (10 mg ml^−1^, Sigma-Aldrich) in ice-cold HBSS (Gibco). Next, spinal cords were extracted, mechanically disassociated and incubated in digestion buffer (collagenase (2 mg ml^−1^, Sigma-Aldrich), dispase (0.2 mg ml^−1^ MP Bio), DNase (0.1 mg ml^−1^, New England), actinomycin D (5 mg ml^−1^, Sigma-Aldrich), triptolide (10 mg ml^−1^, Sigma-Aldrich) and anisomycin (27.1 ml ml^−1^, Sigma-Aldrich)) for 15 min at 0 rpm, and additional 15 min at 300 rpm at 37 °C. After digestion, the tissue suspension was filtered through 100 μm strainer. Next, 9 ml of HBSS were added, and debris was removed from each sample using debris removal solution (Miltenyi Biotec) according to the manufacturer’s protocol. After debris removal, cells were counted, and 16,000 cells per sample were loaded for library preparation.

Before mapping and quantification, the quality of the samples was assessed using fastQC v.0.12.11, applied on raw files; the outputs were summarized using multiQC (v.1.14)^55^. All of the samples were aligned to the GRCm38 (Ensembl 93 from pre-built Cell Ranger reference (v.3.1.0)) reference genome using Cell Ranger (v.7.1.0)^56^, including intronic reads. The resulting feature–barcode raw matrices were loaded in Seurat v.4.3.0.1 and consolidated into one Seurat object. Next, cells were filtered using the following thresholds: 400 < number of genes/features < 8000; 800 < number of UMIs < 35,000; 3% < proportion of reads incident to ribosomal genes < 30%; proportion of reads incident to mitochondrial genes < 5%. All of the thresholds were determined on the basis of distributions of respective values, assessed on violin plots, and default UMAPs. The expression levels were normalized using the LogNormalize method from Seurat with the default parameters. Variable features were found using vst^57^; the top 5,000 most variable features were retained for subsequent steps of the analysis. The expression levels were then scaled and centred. Deterministic PCA (prcomp) was performed on the variable features. The first 50 principal components were retained for subsequent analyses. Harmony^58^ was used to correct batch effects assessed on the Illumina flow cell batches (*θ* = 2). UMAPs were then calculated on the batch-corrected, normalized expression matrix using 50 Harmony dimensions. The optimal parameters for determining partitions/clusters on the given dataset were determined using the ClustAssess pipeline^59^. The shared nearest-neighbour graph was created using FindNeighbors. Clusters were determined using the FindClusters function with the Louvain clustering algorithm, with a resolution parameter of 0.3. The cluster-specific marker genes were determined both using cluster versus complement and all pairwise cluster versus cluster approaches, using the FindMarkers function. The differential expression analysis was performed using the Wilcoxon test, with additional criteria on log_2_[FC] and Benjamini–Hochberg-adjusted *P* value. The enrichment analysis of the marker genes was performed using g:profiler.

For the RNA velocity analysis^24^, loom files were generated using velocyto (v.0.17.17) on all quantified genes. scvelo (v.0.2.5) was used to perform the analysis (that is, filtering, normalization, computing first and second-order moments, and subsequently estimating velocity stochastically). All cluster identification and velocity analyses were performed in R (v.4.2.3) on high-memory Linux servers.

Pyscenic^60^ was used with the metadata made available for the *Mus musculus* (mm10) reference genome, making use of the docker container to generate a loom object from our Seurat object. We used loompy^61^ to create a SCope object, subsequently explored using the SCope web application.

Data-derived filters used for the Nd6 dataset comprise number of genes/features < 2,500; number of UMIs < 5,000; 5% < proportion of reads incident to ribosomal genes < 20%; proportion of reads incident to mitochondrial genes < 15%. For the *Ndufs4* dataset, the filtering thresholds were as follows: number of genes/features < 3,000; number of UMIs < 3,000; 5% < proportion of reads incident to ribosomal genes < 40%; proportion of reads incident to mitochondrial genes < 30%. The *Ndufs4* data were batch-corrected using Harmony^58^ for different pools. For both datasets, ClustAssess^59^ was used to establish the features used and a stable cluster configuration.

For assigning cell types, the outputs of several methods were corroborated.

The unsupervised angle is based on a cluster-driven method relying on stable ClustAssess partitions at a high resolution (that is, a high number of clusters). The smaller clusters were assigned to cell types based on expressed marker genes, which allowed a more granular assessment of transcriptomic signatures. This approach facilitated the reconstruction of assignments for the original, larger clusters. We used this method to assign cell types to the human single-cell data.

The secondary approach was based on a strict cell-centric method for which we ranked each cell based on its median expression of a manually curated, literature-based, gene set associated with one cell type. The cell type was driven by the highest rank; cells with median expression of 0 for all predefined cell types were left unassigned.

The third angle was based on a lenient voting scheme based on a minimum number of genes (all *k* combinations out of the predefined *n* genes were permitted) expressed in any one cell; a minimum average expression value was also used. The implementation of this voting scheme is presented in ClustAssess^59^. For assigning cell identities, we required an average expression higher than 2, and at least 2 marker genes present.

The following marker genes were used: macrophages: *Ms4a7*, *Ccl7*, *Ecm1*, *Mgst*, *Arg1*; microglia: *Serpine2*, *P2ry12*, *Siglech*, *Slc2a5*, *Plxdc2*, *Sall1*, *Sparc*; Monocytes: *Vim*, *Chil3*, *Plac8*, *Ccr2*; dendritic cells: *Flt3*, *H2-DMb2*, *Itgax*, *Ccl17*, *Ccr7*, *Ifitm1*; Neutrophils: *S100a8*, *S100a9*, *Csf3r*, *Cxcr2*, *Mmp9*, *Csf1*, *Il1r2*; CNS-associated macrophages: *Mrc1*, *Lyve1*; T cells: *Cd3e*, *Bcl11b*, *Cd4*, *Cd8a*, *Il2ra*; NK cells: *Ncam1*, *Gzmb*, *Prf1*, *Ifng*.

### Bulk RNA-seq

For comparative gene expression analysis, data obtained from our hiMG differentiation protocol were compared with previously available datasets of primary human microglia isolated from post mortem tissue and microglia derived from human iPS cells^62,63^. The quality checking was performed using fastQC v.0.12.12 on all raw files. The outputs were summarized using multiQC v.1.14. Initial sequencing depths ranged from 21.2 million to 25.7 million reads, aside from MG 3, which had 58.5 million reads. seqtk (v.1.3-r106)^64^ was used for subsampling reads, without replacement to 22 million reads, for the MG 3 sample^65^. Subsequently, all of the samples were aligned to the GRCh38.p13 genome using STAR v.2.7.10a (paired-end mode)^66^. Expression quantification was performed using featureCounts (v.1.6.3)^67^ against the gtf matching the reference *Homo sapiens* genome. Post-quantification quality assessment identified one replicate per sample as an outlier; the subsequent analysis was based on three replicates per sample. Next, noisyR (v.1.0.0)^68^ was used to estimate and remove noise from the count matrix. The filtered raw expression matrix was normalized using quantile normalization^69^, within the bulkAnalyseR framework. DEGs were identified using edgeR^70^ and DESeq2^71^. The *P* values were adjusted using Benjamini–Hochberg multiple-testing correction. The enrichment analysis was performed using all expressed genes, above noise level, as the background set using g:profiler^72^. A Shiny app was generated using bulkAnalyseR (v.1.1.0)^73^ to provide a user-friendly interface for the analysis and visualization of the data.

### Reanalysis of available RNA-seq human datasets

Raw fastQ files from previous studies^27,28^ were downloaded from the European Nucleotide Archive using fasterq-dump. The quality checking and mapping leading to the filtered feature–barcode matrices were performed as described above. For ref. ^28^, cells with a number of genes/feature between 200 and 5,000 were retained; an upper threshold of maximum 20,000 UMI counts was used, and the maximum mt% was set to 5%. For the ref. ^27^ dataset, cells with less than 4,000 maximum number of genes were retained; an upper threshold of 15,000 was used for the maximum UMIs per cell; 5% maximum proportion of fragments incident to mitochondrial genes; 10% maximum proportion of reads incident to ribosomal genes.

Both datasets were normalized using SCTransform. The ref. ^28^ dataset was batch-corrected using Harmony on the patient variable, with *θ* = 2. To find a reliable clustering, on each separate dataset, ClustAssess was used with 20–50 iterations, testing resolution parameters between 0.1 and 1.5 (0.1 increment steps). For the ref. ^27^ dataset, the top 4,500 highly variable features were selected for subsequent analyses. For ref. ^28^ dataset, the top 3,500 highly variable features yielded the most stable results. For both, the resolution parameter was set to 0.6.

For assessing homology, we considered the log-transformed mean expression for homologous genes shared between cluster 4 in our mouse dataset and the MAMS clusters identified using the cluster-based cell-type assignment method. Adjusted *R*^2^ values were computed by fitting a linear model, and we obtained the values 0.40 for ref. ^28^ and 0.38 for ref. ^27^. For identifying enriched gene sets, we used gprofiler^74^ on markers found using a receiver operator characteristic (ROC) test with a log-transformed fold change threshold of 0.25 and a threshold for the minimum percentage of cells expressed of 0.1. All analyses were performed on R v.4.2.3, on high-memory servers.

### Steady-state metabolomics using LC–MS

For ex vivo LC–MS, data analysis was performed as previously described^75,76^. In brief, mice were deeply anaesthetized with i.p. injection of ketamine–xylazine and culled by cervical dislocation. Spinal cords were extracted from the spinal columns using a 5 ml syringe filled with ice-cold sorting buffer (DMEM no phenol red (Thermo Fisher Scientific) + 25 mM HEPES (Sigma-Aldrich) + 5% dialysed FBS (Thermo Fisher Scientific) + 1 mM EDTA (Honeywell Fluka) + 1× GlutaMAX (Thermo Fisher Scientific)). Spinal cords were mechanically dissociated in a glass Dounce tissue homogenizer with 7 ml of cold homogenization buffer (sorting buffer + 10 mg ml^−1^ of DNase). After tissue dissociation, the suspension was filtered through a pre-wet 40 μM strainer and the homogenizer was rinsed with 2 ml of homogenization buffer. The samples were then transferred to 15 ml Falcon tubes and 2.7 ml of 90% Percoll (GE Healthcare) in 10× PBS (Thermo Fisher Scientific) was added to each sample to remove myelin and debris. The 15 ml Falcon tubes were then inverted 10 times gently and the samples were centrifuged at 800*g* for 20 min at 4 °C with a brake speed of 0. Myelin debris visibly layered at the surface was carefully removed with a pipette. Ice-cold buffer (95% autoMACS rinsing solution (Miltenyi Biotec) and 5% MACS BSA (Miltenyi Biotec)) was added to the samples (to fill the 15 ml tubes) and the samples were centrifuged at 800*g* for 5 min at 4 °C to remove the remaining Percoll. The pellets were resuspended in sorting buffer (1 ml + 7 ml) and then further centrifuged at 800*g* for 5 min at 4 °C. Pelleted cells were resuspended in 100 μl of sorting buffer and 0.5% SYTOX blue dead cell stain (Thermo Fisher Scientific).

The samples were sorted using a BD FACS Aria III cell sorter set to yield with an 85 μm nozzle using the FACS gating strategy summarized in the Extended Data Fig. 1b. In brief, the live-cell gates were set based on the unstained WT sample, SYTOX-stained WT sample treated with DMSO (positive control for cell death) and non-TAM-treated *Cx3cr1-YFP*^*creERT2*^*R26*^*tdTomato*^ samples (to control for bleed-through). FACS-sorted cells were collected in polypropylene tubes, centrifuged at 800*g* for 3 min at 4 °C and resuspended in ice-cold PBS 1× (at a concentration of 1 million cells per ml).

An additional 1 ml of PBS 1× was added to each sample and cells were further centrifuged at 800*g* for 3 min at 4 °C. Pelleted cells were then resuspended in cold metabolite extraction buffer (at a concentration of 1 million cells per ml), moved to an autosampler vial and stored at −80 °C. When all of the samples were collected (before LC–MS analysis), autosampler vials were immerged in a methanol dry ice bath twice (10 min in and 5 min out). A total of 10 μl per sample was pulled together to generate a pulled sample control. All of the samples were then stored at −80 °C for LC–MS analysis.

Metabolite extraction was performed at 1 million cells per ml on frozen cell pellets in ice-cold methanol, acetonitrile and water (5:3:1) and vortexed for 30 min at 4 °C. After centrifugation (18,000*g* for 10 min at 4 °C), the supernatants were transferred to prechilled autosampler vials (Phenomenex) and stored at −80 °C for subsequent LC–MS analysis. LC–MS analysis was performed on randomized samples using the Vanquish UHPLC system coupled online to a Q Exactive Orbitrap mass spectrometer (Thermo Fisher Scientific) in positive- and negative-ion modes (separate runs). The LC system was fitted with a Kinetex C18 column (150 mm × 2.1 mm, 1.7 μm; Phenomenex); mobile phases and 5 min gradients were used as previously described in detail^75^. The acquired spectra (.raw) were converted to.mzXML format using RawConverter and the signals were subsequently annotated and integrated using Maven (Princeton University) in conjunction with the KEGG database. Quality-control samples and instrument stability were assessed as described previously^76^.

Analysis of LC–MS data was performed using MetaboAnalyst v.5.0 (https://www.metaboanalyst.ca/). Features with >50% missing values were removed (ADP-ribose, IMP, UDP-glucose and UDP-GlcNAc were also removed as there were too many missing values). The remaining missing values were estimated using *k*-nearest neighbours with no data filtering. Data were normalized to the median, followed by log-transformation and auto scaling to obtain normalized arbitrary units. Partial least squares–discriminant analysis (PLS-DA) was performed on the whole LC–MS dataset to generate a three-dimensional score plot among selected components. A hierarchical clustering heat map was generated on normalized data using autoscale features and the Ward clustering method. Statistical analysis of metabolites found to be significantly altered among all conditions was performed using one-way ANOVA (Graph Pad Prism 9 for macOS, GraphPad) followed by an unpaired *t*-test for multiple comparisons (Supplementary Data 3). Direct comparisons between cells belonging to two different disease stages were performed to generate volcano plots (fold change threshold: 2.0; *P*-value threshold: 0.1). Correlation analysis against a given feature or stage was performed with the PatternHunter tool of MetaboAnalyst using Pearson *r* as distance measure.

### LD-REIMS analysis

Data were first preprocessed using a workflow developed in-house and described in detail previously^77^. Concisely, a signal-to-noise-ratio-based peak detection algorithm was used to reduce the three-dimensional raw data into lists of detected peaks at each pixel. After the effective data resampling, further reduction in data size was achieved through a semi-supervised region of interest (ROI) selection procedure, which performed automatic annotation of pixels to be either sample or background related according to some preliminary manual selection. This ROI mask file generated was then loaded in the nominal next step of the workflow, which aligned and mass corrected all of the detected peaks through a two-step algorithm using user-defined references masses (*m*/*z* = 174.0408, 255.2324, 766.5392 in this case) as tissue-unrelated peaks were filtered by the custom-built SPUTNIK package. As a result, a data cube of dimension *M* × *N* was produced for each imaging run, where *M* is the total number of pixels and *N* is the length of the common mass axis, which was also shared between all runs by considering only the masses that are common in all datasets.

To probe the metabolic differences between varying regions and between images in an untargeted fashion, unsupervised colocalization analysis based on ranked intensity correlations was performed on the preprocessed data. This effectively paired up spectral features that produced statistically significant spatial correlation, of which the relative intensity distributions can be overlayed for visual inspection. All paired features extracted this way were then manually identified through literature search and online databases (HMDB)^78^, then visualized on a circle plot.

To facilitate multivariate supervised analysis, further refinement of the preprocessed data was performed by computing a weighted average of structural similarity index (SSIM) and multi-SSIM for images of every spectral feature with a representative reference image; those that produced a weighted average below a perceptually defined threshold were deemed to be belonging to either background or isotopes and hence removed. The remaining data cube was then visualized by a spectral unmixing algorithm^79^ into (*k* = 2) components (corresponding to tissue and background in an image) of which the abundances can be spatially mapped to give high-contrast images. Spectra from certain anatomically important regions (such as infiltrated white matter, as seen on haematoxylin and eosin staining) were then labelled and extracted by means of manual ROI selection on the abundance image. Weighted logistic regression classification models were then built on the labelled spectra and their performance was assessed using leave-one-out cross-validation between groups. Model refinement through feature selection was then performed based on recursive feature elimination. The validity of the selected features was verified by using them to train another logistic regression model, and then comparing the ROC curves of the new and original models and their respective areas under the curve. These features were considered to be significant in driving the separation between anatomical and pathological features within the imaging data and were identified according to the same procedure as before.

### In vitro microglial cultures

All cell lines were tested for mycoplasma contamination on a regular basis using a PCR-based test and were found to be negative for mycoplasma. BV2 microglia were cultured as previously described^13^. In brief, BV2 microglial cells (gift from A. Tolkovsky) were kept in culture and expanded in expansion medium (DMEM high glucose (Gibco), 2% FBS (Thermo Fisher Scientific), 1% penicillin–streptomycin (Thermo Fisher Scientific)). After reaching 70% confluency, BV2 cells were washed with warm PBS 1×, detached with trypsin-EDTA 0.05% (Thermo Fisher Scientific) for 3 min followed by trypsin neutralization in expansion medium.

Primary microglia were obtained from mixed glial cultures from cerebral cortices from postnatal day 1–3 (P1–P3) WT and *Nd6* unsexed pups. In brief, primary microglia were obtained by peeling off the meninges and cortices were dissected, tissue mechanically dissociated and transferred to the enzyme solution (5 ml Neurobasal-A, Gibco; 6.19 mg papain, Worthington Biochemical) at 37 °C for 15 min. Next, enzyme digestion was stopped by adding microglia medium (DMEM high glucose (Gibco), 10% fetal bovine serum (FBS, Gibco), 1% penicillin–streptomycin (Gibco)), the tissue suspension was then centrifuged at 300*g* for 5 min and the supernatant was removed and incubated in disassociation solution (1 ml Neurobasal-A, Gibco; 5 μl 1 mg ml^−1^ DNase). After 2 min, the reaction was stopped by adding microglial medium, the cell suspension was centrifuged at 300*g* for 5 min, resuspended and plated in a 10 cm Petri dish in microglial medium. The next day, cellular debris was removed, and the mixed-glia culture was incubated in microglial medium with 20% L-929 conditioned medium obtained as previously described^13^. After 2 weeks, microglia were attached to the bottom of the Petri dish, whereas astrocytes formed an upper monolayer. Primary microglial cells were then separated from astrocytes by mild trypsinization, as described previously^80^. After astrocytes were removed, cells were scraped in ice-cold PBS, and centrifuged at 300*g* for 5 min at room temperature. Pelleted cells were resuspended, counted and plated in microglia medium with 20% L-929. Cells were allowed to rest for 48 h before experiments were performed.

hiMGs were derived from human iPS cells, line HPSI0214i-wibj_2. Human iPS cells were cultured in mTeSR1 (Stem Cell Technologies) on hESC-qualified Matrigel-coated plates (Corning). Human iPS eclls were routinely passaged using Accutase cell detachment solution (Stem Cell Technologies). Using a protocol with minor modifications, microglia were differentiated from iPS cells through embryoid bodies (EBs) and primitive macrophage precursors (PMPs)^81,82^. In brief, iPS cells were dissociated to single cells with Accutase cell detachment solution and plated at 10,000 cells per well in 96-well ultra-low attachment plates (Corning) in 100 μl of microglia embryoid body induction media (MEBIM) supplemented with the ROCK inhibitor StemMACS Y27632 (Miltenyi Biotec) before centrifugation at 300*g* for 3 min at room temperature. Plated cells were then stored in a 5% CO_2_ incubator set to 37 °C. EBs were cultured for 4 days with a half medium (that is, 50 μl) exchange with MEBIM occurring after 2 days. When performing the half-medium change, small-molecule concentrations were doubled to account for the medium already present in the wells. After 4 days, 16 EBs were plated per well of tissue-culture treated six-well plates in 3 ml of microglial differentiation medium A (MDM-A). From this point forward, 2 ml medium was exchanged every 5–6 days. When performing the two-third medium change, small-molecule concentrations were doubled to account for the medium already present in the wells. After 14 days, PMPs were collected from suspension during a medium exchange. Collected medium was first filtered through a 40 μm nylon membrane then centrifuged at 500*g* for 5 min at room temperature. Pelleted cells were resuspended in an appropriate volume of microglial differentiation medium B (MDM-B) and a cell count was performed using Trypan Blue (Thermo Fisher Scientific) exclusion. Cells were plated at a density of 180,000–200,000 cells per cm^2^ in six-well plates in 1.5 ml of MDM-B. PMPs adhered to the uncoated tissue culture-treated plastic within 24 h. The maturation of the PMPs into microglia occurred over 14+ days, with half of the medium exchanged every 3 days. When performing the half-medium change, small-molecule concentrations were doubled to account for the medium already present in the wells. All cytokines and growth factors were obtained from Peprotech. After a minimum of 14 days of maturation, microglia-like cells were incubated in 2 ml of Accutase cell detachment solution for 20–30 min in a 5% CO_2_ incubator set to 37 °C. After 20–30 min, any remaining attached cells were detached with gentle pipetting. Microglial-like cells were centrifuged at 500*g* for 5 min at room temperature. Pelleted cells were resuspended in an appropriate volume of MDM-B and a cell count was performed using Trypan Blue exclusion. Cells were allowed to rest for 48 h before experiments were performed.

### In vitro cytotoxicity assay

The cytotoxicity of the small molecules on microglia was measured using the CellTox Green Cytotoxicity Assay (Promega) according to the manufacturer’s instructions. BV2 microglia and primary microglia were seeded at 100,000 cells per well and hiMG at 75,000 cells per well in black 96-well clear-bottom plates in their respective experimental media (BV2: DMEM and 1% penicillin–streptomycin; primary microglia: DMEM, 10% FBS, 20% L-929 medium and 1% penicillin–streptomycin; and hiMG: advanced DMEM/F12, 1% N2 supplement, 1% GlutaMAX, 0.1% 2-mercaptoethanol, 10 ng ml^−1^ GM-CSF, 50 ng ml^−1^ TGFβ1 and 100 ng ml^−1^ IL-34). For BV2 microglia, 24 h after plating the cells were stimulated with LPS (100 ng ml^−1^, serotype EH100, Enzo) and IFNγ (10 ng ml^−1^, Peprotech) for 24 h in experimental medium. For primary microglia and hiMG, the cells were stimulated for 24 h with LPS + IFNγ in experimental medium 48 h after replating. Cells were then treated with either rotenone (1 µM, Sigma-Aldrich), metformin (10 mM, Sigma-Aldrich), dimethyl malonate-DMM (10 mM, Sigma-Aldrich), disodium malonate-DSM (10 mM, Sigma-Aldrich), 4-octyl itaconate-4-OI (125 μM, StemCell Technologies) or S1QEL1.1 (0.1 μM, Caymen Chemical) individually or in combination for 30 min.

After treatment, the medium was removed, and the cells were incubated with Hoechst (1 μg ml^−1^) at room temperature for 10 min followed by fluorescence measurement at 358 nm excitation and 461 nm emission on the Tecan Infinite M200 Pro plate reader. Next, the cell lysis solution was added to the designated wells for 30 min at room temperature as a positive control for cell death. After 30 min, the cells were incubated with the CellTox reagent for 15 min at room temperature and shielded from light. The fluorescence was then measured at 485–500 nm excitation and 520–530 nm emission on the Tecan Infinite M200 Pro plate reader. Cell toxicity was determined by first subtracting the medium-only background fluorescence from the Hoechst and CellTox stains, and the ratio of CellTox fluorescence over Hoechst Fluorescence for each well was calculated. The percentage death was then calculated by taking the ratio of CellTox/Hoechst over the average cell death positive control values and multiplying by 100.

### Assessment of ROS production in vitro and ex vivo

For the in vitro analysis of mitochondrial superoxide production after RET induction, the MitoSOX Red Mitochondrial Superoxide Indicator (Thermo Fisher Scientific) probe was used according to the manufacturer’s instructions. BV2 microglia and primary microglia were replated at 100,000 cells per well and hiMGs at 75,000 cells per well in 96-well black clear-bottom plates in their respective experimental media (as described above). For BV2 microglia, 24 h after plating the cells were stimulated with LPS + IFNγ for 24 h in experimental medium. For primary microglia and hiMG, the cells were stimulated for 24 h with LPS + IFNγ in experimental medium 48 h after replating. For all cell types, 24 h after stimulation the medium was removed, and RET was induced by adding 100 μl of the RET solution (in the cell types’ experimental medium) containing oligomycin (Sigma-Aldrich) at a final concentration of 2 μM and dimethyl succinate at a final concentration of 20 mM (Sigma-Aldrich). After 15 min at 37 °C (5% CO_2_ incubator), 100 μl of MitoSOX (2 μM final concentration) and 20 μl of either rotenone (1 μM), metformin (10 mM), dimethyl malonate-DMM (10 mM), disodium malonate-DSM (10 mM), 4-octyl itaconate-4-OI (125 μM) or S1QEL1.1 (0.1 μM) individually or in combination. After 30 min, the medium was removed from the wells and washed once in 150 μl of 1× HBSS (without Ca^2+^ and Mg^2+^) (Gibco) and then replaced with 150 μl of 1× HBSS (without Ca^2+^ and Mg^2+^). The MitoSOX signal was acquired at 510 nm excitation and 580 nm emission with a Tecan Infinite M200 Pro plate reader.

For the analysis of MitoSOX using flow cytometry, BV2 cells were replated at 0.5 × 10^6^ cells per well in a 6-well plate in 1 ml of experimental media (DMEM (Thermo Fisher Scientific) with 1% penicillin–streptomycin (Thermo Fisher Scientific)). At 24 h after replating, the cells were stimulated for 24 h with LPS + IFNγ in experimental medium. At the end of the 24 h LPS + IFN*γ* treatment timepoint, RET was induced (as described above). After 15 min, rotenone (1 µM) was added to the well. After 30 min, the medium was collected from the well into a 15 ml conical tube. Then, 500 μl of Accutase cell-dissociation reagent (Thermo Fisher Scientific) was added to the well and the plate was incubated at 37 °C for 5 min. After 5 min, 1 ml of 1× PBS was added to the well and the cells were transferred to the 15 ml conical tube with the conditioned medium. The cells were then pelleted at 300*g* for 5 min. The supernatant was removed, and the cell pellet was resuspended in 100 μl of MitoSOX Red working solution (1 μl of a 5 mM stock into 1 ml of 1× PBS). The cell suspension was moved to a 1.5 ml microcentrifuge tube and incubated at 37 °C in an Eppendorf Thermomixer comfort machine set to 500 rpm for 30 min protected from light. After 30 min, labelled cells were pelleted at 300*g* for 5 min. The supernatant was removed, and the cell pellet was resuspended in 300 μl of 1× PBS and transferred to a polystyrene flow cytometry tube (Corning). The samples were kept on ice until acquisition. Then, 10 min before analysis, a working solution of DAPI (1:1,000 in 1× PBS) (Millipore Sigma) was added to the sample (1:100 of working solution).

Flow cytometry gates were set using the FACS-gating strategy summarized in Extended Data Fig. 7b. In brief, gates were set on DAPI- and MitoSOX-Red-positive controls and >200,000 total events were captured using the BD FACS Aria III cell analyser. The FCS files were exported, and analysis was performed using FlowJo v.10 (BD Biosciences).

Ex vivo ROS production of *Cx3cr1-YFP*^*creERT2*^*R26*^*tdTomato*^ mice was obtained after exposing CNS homogenates to 1 μM rotenone or vehicle solution for 30 min, at 37 °C with constant shaking at 500 rpm. We then stained the samples using 5 μM of the CellROX Deep Red Flow Cytometry Assay Kit (Thermo Fisher Scientific) in sorting buffer for 30 min, at 37 °C with constant shaking at 500 rpm. The samples were immediately analysed by flow cytometry on the Aria Fusion (BD Biosciences) system using the FACS gating strategy summarized in the Extended Data Fig. 1b with Sytox Blue (Thermo Fisher Scientific, S34857) to identify live cells. Analysis was performed using FlowJo v.10 (BD Biosciences) in the Sytox-Blue-negative, RFP^+^YFP^+^ population (microglia) and RFP^−^YFP^+^ population (predominantly consisting of infiltrating myeloid cells). Histograms of far-red fluorescence corresponding to the CellROX signal were assessed, and geometric means were extracted.

### Mitochondrial membrane potential in vitro and ex vivo

Cells were replated in their respective experimental medium (described above) in a 96-well black clear-bottom plate (Thermo Fisher Scientific) at a density of 0.5 × 10^5^ cells per well. Then, 24 h after replating BV2 microglia and 48 h after replating hiMG, cells were stimulated with LPS + IFNγ in their respective experimental medium. After 24 h of stimulation, RET was induced as described above. After 15 min at 37 °C, cells were stained with either tetramethylrhodamine methyl ester perchlorate-TMRM (Cambridge Bioscience, final concentration 50 nM, for BV2 microglia) or tetramethylrhodamine ethyl ester-TMRE (Abcam, final concentration 200 nM, for hiMG) and rotenone (1 μM) was added to the wells and the plate was incubated for 30 min at 37 °C. For the TMRM probe, the signal was acquired at 548 nm excitation and 575 nm emission on a Tecan Infinite M200 Pro plate reader. For the TMRE probe, the medium was removed from the wells and the cells were washed twice with 100 μl of 1× PBS/0.2% BSA. After washing, 100 μl of 1× PBS/0.2% BSA was added to the well and the signal was acquired at 549 nm excitation and 575 nm emission on the Tecan Infinite M200 Pro plate reader.

The ex vivo mitochondrial membrane potential of *Cx3cr1-YFP*^*creERT2*^*R26*^*tdTomato*^ mice was obtained by staining brain and spinal cord homogenates using 200 nM of the mitochondrial membrane potential indicator MitoView633 (Biotium) in sorting buffer for 30 min, at 37 °C with constant shaking at 500 rpm. The samples were immediately analysed using flow cytometry on the Aria Fusion (BD Biosciences) system using the FACS gating strategy summarized in the Extended Data Fig. 1b with Sytox Blue (Thermo Fisher Scientific, S34857) to identify live cells. Analysis was performed using FlowJo v.10 (BD Biosciences) in the Sytox-Blue-negative, YFP^+^ and RFP^+^ population (microglia). Histograms of far-red fluorescence corresponding to the MitoView633 signal were assessed, and geometric means were extracted.

The ex vivo mitochondrial membrane potential of CD45^+^Cd11b^+^ myeloid cells from WT and *Nd6* mice was obtained by staining brain and spinal cord homogenates on ice in sorting buffer using Brilliant-Violet-510-conjugated antibodies against CD45 (BD Horizon, 1 μl per 1 million cells), FITC-conjugated antibodies against CD11b (BD Pharmingen, 1 μl per 1 million cells) and Zombie Red (BioLegend, 1:500). The samples were then centrifuged at 500*g* for 5 min at 4 °C. Sorting medium was aspirated, and cells pellets were resuspended and stained in 200 nM of the mitochondrial membrane potential indicator MitoView633 (Biotium) in sorting buffer for 30 min, at 37 °C with constant shaking at 500 rpm. The samples were immediately analysed by flow cytometry on Aria Fusion (BD Biosciences) using the FACS gating strategy summarized in the Extended Data Fig. 9g with Zombie Red (Thermo Fisher Scientific, S34857) to identify live cells. Analysis was performed using FlowJo v.10 (BD Biosciences) in the Zombie-Red-negative, FITC-positive and Brilliant-Violet-510-positive population. Histograms of far-red fluorescence corresponding to the MitoView633 signal were assessed, and geometric means were extracted.

### Microglial cells and SH-SY5Y in vitro co-cultures

SH-SY5Y cells (gift from M. Whitehead) were kept in culture and expanded in growth medium (DMEM-F12 (Gibco), 10% FBS, 1% penicillin–streptomycin). The growth medium was refreshed every 4 to 7 days. After reaching 80–90% confluency, SH-SY5Y cells were washed with warm PBS (Gibco), detached with trypsin-EDTA 0.05% (Thermo Fisher Scientific) for 3 min, trypsin neutralized in growth medium and centrifuged at 400*g* for 5 min. Next, SH-SY5Y cells were counted and seeded at a density of 5 × 10^4^ cells per well in 12-well plates with differentiation medium (Neurobasal medium (Thermo Fisher Scientific) 2% B27 supplement (Thermo Fisher Scientific), 1% GlutaMAX (Thermo Fisher Scientific) and 10 μM all-*trans* retinoic acid (StemCell Technologies)). Differentiation medium was replaced every other day until day 9 after seeding for co-culture experiments with BV2 microglia, primary microglia and hiMGs.

BV2 microglia, primary microglia and hiMGs were maintained and cultured as described above. To time the co-cultures, on day 8 (24 h before stimulation) of the SH-SY5Y differentiation, BV2 microglia were replated inside a 0.4-μm-pore size Transwell insert (12 well-size, Millipore) at a density of 1 × 10^5^ cells per Transwell with 1 ml of experimental medium. Transwell inserts were placed in a 12-well plate prefilled with 1 ml of experimental media per well. For primary microglia and hiMG, the cells were replated inside the 0.4-μm-pore size Transwell insert on day 7 (48 h before stimulation) of the SH-SY5Y differentiation. On day 9 of the SH-SY5Y differentiation, the BV2 microglia, primary microglia, and hiMG were stimulated with LPS/IFNγ in their respective experimental media for 12 h.

For all microglial cells, 12 h after the LPS/IFN*γ* stimulation (day 10 of the SH-SY5Y cell differentiation) RET was induced (as described above) in the Transwell for 15 min at 37 °C. After 15 min rotenone (1 μM) was added to the Transwell. The cells were further incubated for 30 min at 37 °C. After 30 min, the medium was removed from the Transwells with the microglia and subsequently transferred to a 12-well plate prefilled with 1 ml of 1× PBS. The Transwell inserts were washed once with prewarmed 1× PBS and put into co-culture with the SH-SY5Y cells. Then, 1 ml of experimental medium was added to the Transwell. After 6 h of co-culture, the Transwells were removed and 10× phase-contrast images of 3–5 ROIs per well were acquired using an Echo Rebel microscope (Discover Echo). After image acquisition, the cells were washed 1× in PBS and 350 μl of RLT lysis buffer (QIAGEN) was added to each well. The plates were stored at −70 °C until RNA isolation.

For the mitoTEMPO experiments, the SH-SY5Y cells were pretreated with 50 μM of mitoTEMPO 12 h before being placed into co-culture with BV2 microglia. For the S1QEL1.1 experiments, BV2 microglia were treated for 30 min with 0.1 μM of S1QEL1.1 after 15 min of RET induction before being placed into co-culture with the SH-SY5Y cells.

For neurite length analysis, the images were converted to 8-bit and contrast was adjusted so the neurites were easily visible using Fiji v.2.0.0. software. Data were quantified by semi-automatic tracking using the NeuronJ plugin from >15 neurite lengths per ROI.

For the caspase-3 experiments, SH-SY5Y cells were seeded at a density of 5 × 10^4^ cells per well on coverslips in a 24-well plate with differentiation medium. Differentiation medium was replaced every other day until day 9 after seeding for co-culture experiments. On day 8 (24 h before stimulation) of the SH-SY5Y differentiation BV2 microglia were replated inside a 0.4-μm-pore size Transwell insert (24-well size, Millipore) at a density of 1 × 10^5^ cells per Transwell with 500 μl of experimental medium. Transwell inserts were placed into a 24-well plate prefilled with 500 μl of experimental medium per well. On day 9 of the SH-SY5Y differentiation, the BV2 microglia were stimulated with LPS + IFNγ in experimental medium for 12 h. Then, 12 h after the LPS/IFN*γ* stimulation (day 10 of the SH-SY5Y cell differentiation), RET was induced (as described above) in the Transwell for 15 min at 37 °C. After 15 min, rotenone (1 μM) was added to the Transwell. The cells were further incubated for 30 min at 37 °C. After 30 min, the medium was removed from the Transwells and subsequently transferred to a 24-well plate prefilled with 500 μl of 1× PBS. The Transwell inserts were washed once with prewarmed 1× PBS and put into co-culture with the SH-SY5Y cells. Then, 500 μl of experimental medium was added to the Transwell. After 6 h of co-culture, the Transwells were removed and the SH-SY5Y cells were washed once with 1× PBS and subsequently fixed with 4% paraformaldehyde for 10 min, washed twice with 1× PBS and blocked for 1 h at room temperature in blocking buffer (0.1% Triton X-100 in 1× PBS (PBS-T) with 10% normal goat serum (NGS)). Coverslips were then incubated at 4 °C overnight with antibodies against cleaved caspase-3 (Cell Signaling, 1:1,000) in antibody blocking buffer (PBS-T with 1% NGS). Next, the coverslips were washed three times with PBS-T for 5 min, incubated with the appropriate secondary antibody (1:1,000) in antibody blocking buffer for 1 h at room temperature and then washed with PBS three times for 5 min. Lastly, the coverslips were counterstained with DAPI for 5 min then mounted onto glass slides with ProLong diamond antifade mountant (Thermo Fisher Scientific) and sealed with nail polish. Images were taken on the Leica DMI400B microscope of three ROIs per coverslip using a ×63 objective. Images were batch analysed using Fiji; the pipeline included fluorescent signal identification by minimum error thresholding followed by the measurement of the mean grey value. The values from three ROIs were averaged for each coverslip, and the resulting values were again averaged to generate the mean fluorescence intensity of each condition.

Catalase gene expression analysis was performed using RT–qPCR. At the end of co-culture experiment, total RNA from SH-SY5Y cells was collected by washing cells with ice-cold 1× PBS and adding 350 μl of RLT lysis buffer (QIAGEN). The samples were then stored at −80 °C until extraction. Total RNA from SH-SY5Y cells was extracted using the RNeasy Mini Kit (QIAGEN) according to the manufacturer’s instructions. For RT–qPCR analysis, equal amounts of RNA were reversed transcribed using the high-capacity cDNA reverse transcription kit (Thermo Fisher Scientific) according to the manufacturer’s instructions. cDNA was then quantified using the NanoDrop 2000c instrument (Thermo Fisher Scientific) and RT–qPCR was performed using the TaqMan Fast Universal PCR Master Mix (2×) (Thermo Fisher Scientific) and TaqMan Gene Expression Assays for catalase (Thermo Fisher Scientific, Hs00156308_m1), and 18S (Thermo Fisher Scientific) was used for normalization. The samples were run in triplicates using the 7500 Fast Real-Time PCR System (Applied Biosystems) and analysed using the ${2}^{-\Delta \Delta {C}_{{\rm{t}}}}$ method.

### Western blot analysis of OXPHOS complexes

Ex vivo isolated microglia were homogenized in 1× RIPA buffer (Abcam) supplemented with 1× protease and phosphatase inhibitors (Thermo Fisher Scientific). Equal protein amounts (5 µg) were resolved by SDS–PAGE on 4–15% Mini-PROTEAN TGX Stain Free gels (30 μl, 10-well comb, Bio-Rad, 4568083) and transferred to polyvinylidene fluoride membranes using the Trans-Blot Turbo Transfer Pack (Bio-Rad, 1704156). The membranes were blocked with 5% milk in 0.1% TBST for 1 h at room temperature. The membranes were then immunoblotted over night at 4 °C with the indicated primary antibodies diluted in blocking buffer (SDHA (Abcam, 1:1,000), total OXPHOS Rodent WB Antibody Cocktail (Abcam, 1:500), NDUFAF1 (Abcam, 1:10,000) and TOMM20 (Santa Cruz, 1:2,000)). The membranes were washed three times with TBST for 5 min and immunoblotted with the appropriate HRP-conjugated secondary antibodies: anti-rabbit (Cell Signaling, 7074, 1:2,000) or anti-mouse (Cell Signaling, 7076, 1:2,000). Membranes were washed three times with TBST for 5 min, then imaged on the ChemiDoc MP Imaging System (Bio-Rad), soaked in Clarity Western ECL Substrate (1705061). Uncropped and unprocessed scans of the blots are provided in the Extended Data Fig. 6.

Protein band densitometry was measured in ImageJ. An identical selection frame was used to determine the mean grey value (MGV) measurement of protein bands and background areas above and below each band. Band intensity (BI) was determined using the formula: BI = (255 − MGV protein band) − [(255 − MGV background above) + (255 − MGV background below)]/2, and values were normalized to TOMM20.

### Mitochondrial DNA copy number

We isolated 30,000 cells per sample using the FACS gating strategy summarized in the Extended Data Fig. 1b and centrifuged them at 500*g* for 5 min at 4 °C. The sorting medium was aspirated, and cells pellets were used for total DNA isolation using the DNeasy Blood & Tissue Kit (Qiagen) according to manufacturer’s instructions. Total DNA was then quantified using the NanoDrop 2000c instrument (Thermo Fisher Scientific). For each sample, 10 ng of DNA was loaded into qPCR reactions using the TaqMan Fast Universal PCR Master Mix (Thermo Fisher Scientific, 4352042) and primers for mitochondrial-genome-encoded MT-ND1 (Thermo Fisher Scientific, 4331182, Mm04225274_s1). TaqMan Copy Number Reference Assay Tfrc (Thermo Fisher Scientific, 4458366) primers were loaded into the same reactions for normalization. qPCR was run on the QuantStudio 7 Flex (Thermo Fisher Scientific, 4485701) system, and the resulting files were analysed using the Quant Studio Design & Analysis Software v.2.5.1 using the ${2}^{-\Delta \Delta {C}_{{\rm{t}}}}$ method.

### Metabolic flux analysis with Seahorse

Metabolic flux measurements were performed on myeloid cells isolated ex vivo from *Cx3cr1-YFP*^*creERT2*^*R26*^*tdTomato*^ mice and on primary WT and *Nd6* microglia cultured in vitro.

For the ex vivo metabolic flux analysis, a Seahorse calibration plate was rehydrated with calibration buffer (Agilent) overnight in a non-CO_2_ incubator at 37 °C the day before FACS isolation of cells. On the day of FACS, the Seahorse cell culture plate was coated with Cell-Tak (Corning) and washed with distilled H_2_O. After FACS (using the FACS gating strategy summarized in the Extended Data Fig. 1b), cells were collected in polypropylene tubes, centrifuged at 800*g* for 3 min at 4 °C and resuspended in 1× PBS (at a concentration of 1 million cells per ml). Cells were then centrifuged at 800*g* for 3 min at 4 °C and resuspended at 1 million cells per ml in sorting buffer. Cells were seeded on the Seahorse XFe96 plate at 40,000 cells per well (up to six replicates per mouse per cell type) and centrifuged at 200*g* for 1 min with 0 brake. The culture plate was placed in an incubator at 37 °C for 30 min and then transferred into a non-CO_2_ incubator for 30 min. The plate was calibrated, and the cells were washed once with mitochondrial assay solution (MAS, made of sucrose 70 mM + mannitol 220 mM + KH_2_PO_4_ 10 mM + HEPES 2 mM + EGTA 1 mM, in distilled H_2_O at pH 7.2). Just before loading the plate into the Seahorse XFe96 analyser, cells were put in MAS + 0.2% BSA + 1 nM plasma membrane permeabilizer (Agilent) + 4 mM ADP (pH 7.4). The cell plate was equilibrated, and the baseline was measured. Glutamate (17.5 mM) and malate (17.5 mM) were used to activate complex I. Rotenone (3 µM, well final concentration) was used to inhibit complex I and disodium succinate (10 mM, well final concentration) (Sigma-Aldrich) was used to activate complex II. Antimycin A (4 µM, well final concentration) was used to inhibit complex III and stop mitochondrial respiration. Five reads were obtained (every 4.63 s) at the baseline and after each injection. If cells did not respond to the addition of antimycin A, they were excluded from further analysis. After the Seahorse run, the cells were fixed with 4% PFA for 10 min and stained with DAPI for 2 min. Using the Leica microsystems CTR4000 system to take images at ×10, the OCR values of every plate were normalized based on the mean area of DAPI of four regions of interest. Seahorse analysis was performed using Wave v.2.6.1. Complex I activity was calculated as the percentage change decrease in the mean OCR obtained from two measurements before and after rotenone injection. Complex II activity was calculated as the percentage change decrease in the mean OCR obtained from two measurements before and after succinate injection.

For the in vitro metabolic flux analysis, primary microglia were replated at 100,000 cells per well of a Seahorse XFe24 Microplate in microglia medium and incubated for 48 h to allow the cells to adhere and rest. After 48 h, the cells were stimulated with LPS + IFNγ in experimental medium for 24 h. The day before the assay, a Seahorse sensor cartridge was rehydrated with calibration buffer (Agilent) overnight in a non-CO_2_ incubator at 37 °C. In each microplate, four wells did not contain cells to serve as background wells, according to the manufacturer’s guidelines. On the day of the Seahorse assay, assay medium (XF DMEM (Agilent) supplemented with XF glucose (10 mM, Agilent), XF glutamine (2 mM, Agilent) and XF pyruvate (1 mM, Agilent)) was prepared and incubated in a bead bath set to 37 °C 1–2 h before the assay. After 24 h of stimulation with LPS + IFNγ, the medium was removed from the microplate containing the primary microglia and replaced with 500 μl of assay medium then placed into a non-CO_2_ incubator for 45 min. The drugs were then loaded into the ports of the sensor cartridge: port A contained oligomycin to inhibit mitochondrial ATP production (1 µM final concentration in well); port B contained FCCP to induce mitochondrial uncoupling and maximal mitochondrial respiration (1 µM final concentration in well); and port C contained rotenone/antimycin A to inhibit mitochondrial complex I and complex III, respectively, and therefore stop mitochondrial respiration (1 µM final concentration in well). The plate was then placed into the Seahorse XFe24 Analyzer to calibrate. After 45 min, the cells were then subjected to the XFe Cell Mito stress test (Agilent) according to the manufacturer’s instructions. The cell plate was equilibrated, and the baseline was measured. Three reads were obtained (every 8.75 min) at the baseline and after each drug injection. If cells did not respond to the addition of drugs, they were excluded from further analysis. After completion of the assay, the medium was removed and 50 μl of RIPA buffer (10× RIPA (Abcam), 100× protease/phosphatase inhibitors (Sigma-Aldrich), distilled H_2_O (Corning)) was added to each well. The protein amount of each well was determined using the Pierce BCA Protein Assay for normalization. Analysis was performed using the Wave v.2.6.1. software (Agilent).

### Genomic PCR and RT–qPCR

The genotype of the mice was verified using genomic PCR by collecting the ear notch and extracting DNA by adding DNA Releasy (15 µl, Anachem) to each sample. The samples were lysed in a thermal cycler: 65 °C for 15 min, 96 °C for 2 min, 65 °C for 4 min, 96 °C for 1 min, 65 °C for 1 min, 96 °C for 30 s, 4 °C on hold and molecular-biology-grade water (120 µl, Thermo Fisher Scientific) was added to each sample. A total of 20–100 ng of DNA from each sample was added to a master mix containing molecular-biology-grade water (7.5 µl) and BioMix Red (12.5 µl, Bioline) with the appropriate amount of primers. The primer sequences for *Cx3cr1*^*cre*^ mice were as follows: C: 5′-AAGACTCACGTGGACCTGCT-3′; mt, R: 5′-CGGTTATTCAACTTGCACCA-3′; WT, R: 5′-AGGATGTTGACTTCCGAGTTG-3′; expected product: mutant, 300 bp; heterozygote, 300 bp and 695 bp; WT 695 bp. The primer sequences for *R26*^*tdTomato*^ mice were as follows: mt F: 5′-CTGTTCCTGTACGGCATGG-3′; mt R: 5′-GGCATTAAAGCAGCGTATCC-3′; WT F: 5′-AAGGGAGCTGCAGTGGAGTA-3′; WT R: 5′-GGCATTAAAGCAGCGTATCC-3′; expected products: mutant, 196 bp; heterozygote, 297 bp and 196 bp; WT, 297 bp. The primer sequences for *Nd6* mice were as follows: F: 5′-TACCCGCAAACAAAGATCACCCAG-3′; R: 5′-TTAGGCAGACTCCTAGAAGG-3′. Primer sequences for *ndufs4* mice were as follows: C loxP_A: 5′-AGCCTGTTCTCATACCTCGG-3′; mt R loxP_B: 5′-GCTCTCTATGAGGGTACAGAG-3′: WT R loxP_C: 5′-GGTGCATACTTATACTACTAGTAG-3′; expected products: mutant, 200 bp; heterozygote 200 bp and 150 bp; WT, 150 bp. Amplification reaction parameters were as follows: initial denaturation at 94 °C for 4 min (1×); denaturation 94 °C for 30 s, annealing temperature 65 °C for 30 s, elongation time 72 °C for 40 s (repeated 35×); final extension 72 °C for 8 min, and 4 °C on hold. The same PCR protocol was applied for the genomic analysis of cells isolated by FACS from *Cx3cr1-YFP*^*creERT2*^*Ndufs4*^*flox/flox*^ mice, where DNA was extracted using the DNeasy Blood & Tissue Kit (Qiagen) according to the manufacturer’s instruction. Next, amplified nucleic acids (1 μl) were mixed with master mix (24 μl, see above), and loaded onto a 3% agarose gel (agarose (Bioline), Tris-buffered saline TAE (Sigma-Aldrich), red nucleic acid gel stain (Biotium)) with a TrackIt 100 bp DNA adder (Thermo Fisher Scientific), and run at 90 V for 60 min in 1× TAE electrophoresis buffer. After the run was completed, DNA bands were visualized with ultraviolet light (312 nm) using the ChemiDoc Imaging System (Bio-Rad) and directly photographed.

For gene expression analysis using RT–qPCR, total RNA from primary microglia and SH-SY5Y samples was collected at a given timepoint. Next, cells were washed with 1× PBS, 350 μl of RLT lysis buffer (Qiagen) was added and the samples were stored at −80 °C until extraction. All in vitro samples for RT–qPCR experiments were processed using the RNeasy Mini Kit (Qiagen) according to the manufacturer’s protocol. For RT–qPCR analysis, equal amounts of RNA were reversed-transcribed using the high-capacity cDNA reverse transcription kit (Thermo Fisher Scientific) according to the manufacturer’s instructions. TaqMan Fast Universal PCR Master Mix (2×) (Thermo Fisher Scientific) and TaqMan Gene Expression Assays was used for *Il1b* (Thermo Fisher Scientific, Mm00434228_m1), *Il6* (Thermo Fisher Scientific, Mm00446190_m1), *Tnf* (Thermo Fisher Scientific, Mm00443258_m1), *Inos* (Thermo Fisher Scientific, Mm00440502_m1) and *CAT* (Thermo Fisher Scientific, Hs00156308_m1). For normalization, mouse *Actb* or 18S were used (Thermo Fisher Scientific). The samples were run in technical duplicates or triplicates using the 7500 Fast Real-Time PCR System (Applied Biosystems) and analysed using the ${2}^{-\Delta \Delta {C}_{{\rm{t}}}}$ method.

### Ex vivo NDUFS4 protein analysis

To determine the ex vivo NDUFS4 protein expression in CX3CR1^+^ cells, brain and spinal cord tissue homogenates were prepared for FACS staining as described in the ‘Steady-state metabolomics using LC–MS’ section (up to obtaining unlabelled cells). The unlabelled cell suspension was then stained with a CD16/CD32 Fc blocking antibody (BD Biosciences, 567021, 1:50) for 10 min on ice. After incubation, 1 ml of cell staining buffer (BioLegend, 420201) was added and the cells were centrifuged for 5 min at 400*g*. The supernatant was then discarded, and the cell pellet was resuspended in 100 μl of cell staining buffer containing antibodies against the cell surface markers CD45 (BD Biosciences, 563891, 1:100, BV510) and CX3CR1 (BioLegend, 149005, 1:500, PE) and the Zombie violet live/dead stain (BioLegend, 423113, 1:100). The cells were incubated for 40 min at 4 °C with gentle agitation of the cells occurring every 10 min. After 40 min, the cells were washed once with 1 ml of cell staining buffer and centrifuged for 5 min at 400*g*. The supernatant was discarded and 500 μl of 1× fix/perm buffer (BD Biosciences, 562574) was added and the cells were incubated for 40 min at 4 °C with gentle agitation of the cells occurring every 10 min. After the cells were fixed, they were washed twice with 1× perm/wash buffer (BD Biosciences, 562574) and centrifuged for 5 min at 400*g*. After the last wash, the supernatant was discarded, and the cell pellet was resuspended in 100 μl of 1× perm/wash buffer containing the antibody against the intracellular marker NDUFS4 (Abcam, ab137064, 1:62.5). The cells were incubated for 40 min at 4 °C with gentle agitation of the cells occurring every 10 min. After 40 min, the cells were washed once with 1 ml of 1× perm/wash buffer and centrifuged for 5 min at 400*g*. The supernatant was discarded, and the cell pellet was resuspended in 100 μl of 1× perm/wash buffer containing the Alexa Fluor 647 secondary antibody (Thermo Fisher Scientific, A21244, 1:100). The cells were incubated for 40 min at 4 °C with gentle agitation of the cells occurring every 10 min. After 40 min, the cells were washed once with 1 ml of 1× perm/wash buffer and centrifuged for 5 min at 350*g*. The supernatant was discarded, and the cell pellet was resuspended in 500 μl of cell staining buffer and immediately analysed on the Aria Fusion flow cytometer (BD Biosciences). Gates were set using the FACS gating strategy that is summarized in the Extended Data Fig. 10c. In brief, unstained single cells were used to gate the negative population; single stains against CD45, CX3CR1 and NDUFS4 were used to gate the positive populations; a single stain against Zombie violet was used to gate the live cell population (negative). Samples were processed and 20,000–50,000 total events were captured. Analysis was performed using FlowJo v.10 (BD Biosciences).

### Small molecules for in vivo studies

The optimal route of administration of DMM (Sigma-Aldrich) and disodium malonate (DSM) (Sigma-Aldrich) that would lead to the lowest blood concentration and highest CNS tissue concentration was determined using a pharmacokinetics study. EAE mice at 1 week (7 days) from clinical disease onset were separated into six treatment groups (*n* = 2 per group): (1) i.p. DMM (160 mg per kg); (2) i.p. DSM (160 mg per kg); (3) oral (drinking water) DMM (1.5 w/v%); (4) oral DSM (1.5 w/v%); (5) i.p. saline; and (6) oral saline. i.p. injections: mice received 5 daily i.p. injections of DMM, DSM or saline, were allowed to rest for 30 min, then the blood was collected through the tail vein. For oral administration, mice were given 5 days of ad libitum access to DMM-, DSM- or saline-treated water; blood was collected through the tail vein daily for 4 days. Obtained blood was centrifuged at 10,000*g* for 5 min and the purified plasma was collected and stored at −80 °C until analysis. At the end of the treatment period, the brain and spinal cord was extracted from the mice and flash-frozen in liquid nitrogen. The frozen tissue was then stored at −80 °C until analysis.

Malonate from blood and tissue was quantified as previously described^83^. In brief, 20–30 mg of tissue was homogenized in 25 μl mg^−1^ extraction buffer (50% methanol, 30% acetonitrile and 20% water) and 1 nmol ^13^C_3_-malonate as an internal standard, using prefilled bead mill tubes (Thermo Fisher Scientific) in a Precellys 24 homogenizer (Bertin Instruments) (6,500 rpm; twice for 15 s, cooling on ice in between). Then, 10 μl blood was added to 450 μl extraction buffer containing the internal standard in a microcentrifuge tube and vortexed. All of the samples were subsequently incubated at −20 °C for 1 h before centrifugation at 17,000*g* for 10 min at 4 °C. The supernatant was transferred to a microcentrifuge tube and centrifuged under the same conditions. The resulting supernatant was transferred to precooled MS vials and stored at −80 °C until analysis by LC–MS/MS.

At 1 week from the onset of clinical signs of EAE, mice were randomized to receive treatment or control substances. These included: saline 0.9%, metformin (Sigma-Aldrich, 100 mg per kg), 4-OI (StemCell Technologies, 50 mg per kg), DMM (Sigma-Aldrich, 160 mg per kg) or DMM (160 mg per kg) + metformin (100 mg per kg). Mice were given daily i.p. injections with the treatment or control substances until the end of the study (30 days after immunization).

### Mouse and human tissue pathology

At the end of the study period, EAE mice were deeply anesthetized with an i.p. injection of ketamine (10 mg ml^−1^, Boehringer Ingelheim) plus xylazine (1.17 mg ml^−1^, Bayer) and transcardially perfused with saline (0.9% NaCl + 5 M EDTA) for 5 min followed by 4% PFA for an additional 5 min. The spinal columns were isolated and post-fixed in 4% PFA at 4 °C overnight. The next day, the spinal cords were extracted, washed in 1× PBS and immersed in a 30% sucrose (in PBS) cryoprotectant solution for at least 72 h at 4 °C. The spinal cords were then embedded in optimum cutting temperature medium in a plastic mould and frozen on 2-methylbutane (Thermo Fisher Scientific) on dry ice. The tissue blocks were cryo-sectioned (30 μm for *Cx3cr1-YFP*^*creERT2*^*R26*^*tdTomato*^ EAE mice, 10 μm for all of the other EAE experiments) using a cryostat (Leica, CM1850) with a microtome blade (Feather) onto Superfrost Plus slides (Thermo Fisher Scientific). The sections were stored at −80 °C until use.

Human tissue was obtained from the Multiple Sclerosis and Parkinson’s Tissue Bank (Imperial College London) under ethical approval (IRAS reference: 279989). Autopsy flash-frozen brain tissue from one case with secondary progressive MS (44 years old, male) was cryo-sectioned with a 10 μm thickness using a cryostat (CM1850, Leica) with a microtome blade (A35, Feather). The sections were stored at −80 °C until use.

### IHC and Bielschowsky staining

For the RFP and YFP double immunohistochemistry (IHC), sections were washed with 1× PBS + 0.3% Triton X-100 (Sigma-Aldrich) and incubated in Bloxall (Vector Laboratories) for 10 min, washed three times for 5 min in 1× PBS + 0.3% Triton X-100, then blocked in 1× PBS + 0.3% Triton X-100 + 10% NGS (Abcam) for 1 h. Antibody incubation was performed overnight at 4 °C with anti-RFP antibodies (rabbit, 1:400, Abcam) in PBS + 0.3% Triton X-100 + 10% NGS. The next day, the sections were washed three times for 5 min in 1× PBS + 0.3% Triton X-100 and incubated with biotinylated goat anti-rabbit (1:1,000, Abcam) for 10 min, washed three times for 5 min in 1× PBS + 0.3% Triton X-100, and incubated with avidin biotin complex (ABC, Vector Laboratories) for 30 min according to the manufacturer’s instructions. After washing three times for 5 min in 1× PBS + 0.3% Triton X-100, the RFP staining was visualized using brown 3,3′-diaminobenzidine (DAB) (Vector Laboratories). The sections were then washed in H_2_O for 5 min, and then once for 5 min in 1× PBS. The sections were put in Bloxall for 10 min, washed three times for 5 min in 1× PBS + 0.3% Triton X-100, blocked with avidin and then biotin, washed three times for 5 min in 1× PBS + 0.3% Triton X-100, and then blocked with 1× PBS + 0.3% Triton X-100 with 10% NGS for 1 h. Antibody incubation was performed overnight at 4 °C with an anti-YFP antibody (chicken, 1:1,000, Abcam). The next day, the sections were incubated with biotinylated goat anti-chicken (1:1,000, Vector Laboratories) antibodies for 1 h, washed three times for 5 min in 1× PBS + 0.3% Triton X-100 and incubated with ABC for 30 min. After three 5 min washes in 1× PBS + 0.3% Triton X-100, the YFP staining was visualized with blue/grey DAB (Vector Laboratories). The sections were counterstained with haematoxylin (Sigma-Aldrich) for 30 s and washed with distilled H_2_O. The sections were covered with mounting medium (Ibidi) and a cover slip placed over top (Thermo Fisher Scientific). Images were taken using the Olympus BX53 system and post-processed using Fiji v.2.0.0.

To quantify the amount of oxidative stress in EAE mice using IHC, we analysed GP91-PHOX expression in the spinal cord of EAE mice at 50 days after immunization^26^. In brief, the sections were air dried at room temperature for 25 min, rinsed with 1× PBS and blocked with Bloxall for 10 min at room temperature, washed three times for 5 min with 1× PBS and incubated using the M.O.M. Immunodetection Kit (Vector Laboratories) according to the manufacturer’s instructions. Next, the sections were incubated overnight at 4 °C with anti-GP91-PHOX antibodies (mouse, 1:200, BD Biosciences) diluted in 1× PBS + 0.3% Triton X-100 with 1% NGS. The next day, the sections were washed three times for 5 min in 1× PBS and incubated with a biotinylated goat anti-mouse secondary antibodies (1:1,000, Thermo Fisher Scientific) for 1 h at room temperature, washed again three times for 5 min and incubated with the ABC kit (Vector Laboratories) for 1 h at room temperature according to the manufacturer’s instructions. After washing three times for 5 min in 1× PBS + 0.3% Triton X-100, staining was visualized by applying DAB (Vector Laboratories). The sections were next washed twice in distilled H_2_O for 5 min. Finally, the sections were dehydrated using an alcohol gradient: 70% (twice for 2 min), 90% (twice for 2 min), 100% (twice for 2 min) and xylene (twice for 2 min), mounted (Ibidi) and coverslipped (Thermo Fisher Scientific).

For IHC analysis of human post mortem brain tissue, the sections were washed with cold methanol and H_2_O_2_ to eliminate endogenous peroxidase activity. After blocking with 10% NGS in PBS solution, the sections were incubated overnight at 4 °C with primary antibodies against PLP (mouse, 1:200, BioRad) and MHC-II (mouse, 1:1,000, BioLegend) in 1.25% NGS in PBS. The next day, the sections were incubated for 1 h and 30 min at room temperature with polymer-based secondary antibodies (Vector Labs). Subsequent reaction product was developed using 3,3′-diaminobenzidine (Vector Labs). The sections were counterstained with haematoxylin, sealed with Pertex Mounting Medium (CellPath) and imaged using the Olympus BX53 microscope with a motorized stage and the Neurolucida software (v.11.07 64-bit, Microbrightfield). White-matter lesions were staged using a robust, previously validated lesion classification system^84–86^. In brief, the CAL was staged according to its neuroanatomical location, cellular density, degree and pattern of demyelination and lesion immunological status. Using these criteria, CAL was defined as a sharply demarcated demyelinating plaque with high MHC-II reactivity specific to the lesion rim, accompanied by sparse myeloid activation within the periplaque.

To assess in vivo axonal loss, spinal cord sections were analysed after Bielschowsky staining, as previously performed^13^. In brief, the sections were rehydrated in distilled H_2_O for 10 min at room temperature and placed into the silver nitrate solution (AgNO_3_) (10%, Sigma-Aldrich) for 20 min. Next, the sections were washed in distilled H_2_O and incubated with ammonium silver solution (AgNO_3_ (10%) with NH_3_OH (32%, Sigma-Aldrich) added until the precipitate became clear) for 20 min at room temperature. After the sections turned brown, the tissue was incubated in NH_3_OH solution (0.05%, Sigma-Aldrich) for 5 min at room temperature and developed in the ammonium silver solution supplemented with 100 μl of developer solution (formaldehyde, 37%, (Thermo Fisher Scientific), citric acid (Sigma-Aldrich) and nitric acid (70%, Thermo Fisher Scientific) in distilled H_2_O). After 2 min of staining development, the sections were quickly incubated in the 0.05% NH_3_OH solution for 5 min and washed three times for 5 min in 1× PBS. Lastly, the sections were dehydrated using an alcohol gradient: 70% (twice for 2 min), 90% (twice for 2 min), 100% (twice for 2 min) and xylene (twice for 2 min), mounted (Ibidi) and coverslipped (Thermo Fisher Scientific).

Quantification of oxidative stress via IHC for GP91-PHOX and axonal loss via Bielschowsky staining in EAE was obtained from analysing *n* ≥ 12 from spinal cord axial sections per *n* ≥ 4 biological replicates positive DAB area and axonal loss area respectively of equally spaced axial spinal cord sections outlined using the Olympus BX53 microscope with a motorized stage and Neurolucida software (v.11.07 64-bit, Microbrightfield). Data are expressed as either the percentage of damaged tissue or the area of positive (%) DAB staining per section (±s.e.m.).

### Immunofluorescence analysis

For the assessment of cell markers and axonal degeneration using immunofluorescence, slides were dried and washed with distilled H_2_O and 1× PBS for 5 min each. Tissue was blocked with 1× PBS + 0.3% Triton X-100 + 10% NGS or NDS for 1 h at room temperature. For those sections stained with primary antibodies made in mouse, slides were blocked with M.O.M mouse IgG blocking reagent (Vector Laboratories) in TBS + 0.1% Tween-20 + two drops of IgG diluent for 1 h at room temperature, followed by a block with M.O.M. protein diluent solution for 5 min at room temperature. The following primary antibodies were used (diluted in 1× PBS + 1% NGS or NDS and kept overnight at 4 °C): anti-GFP (chicken, 1:1,000, Abcam), anti-IBA1 (rabbit, 1:500, FUJIFILM Wako; or goat, 1:500, Novus biologicals), anti-CD3 (rat, 1:500, BD bioscience), anti-CD19 (rabbit, 1:500, Abcam), anti-GFAP (mouse, 1:400, Sigma-Aldrich) anti-CX3CR1 (rabbit, 1:300, Novus biologicals), anti-NEUN (mouse, 1:400, Sigma-Aldrich), anti-OLIG2 (goat, 1:500, Novus biologicals), anti-SPP1 (goat, 1:500, R&D Systems), anti-GP91-PHOX (rabbit, 1:500, Proteintech; or mouse, 1:200, BD bioscience) anti-CASPASE3 (mouse, 1:600, Novus biologicals), anti-NDUFS4 (mouse, 1:100, Abcam; or rabbit, 1:500, Novus biologicals), anti-amyloid precursor protein-APP (mouse, 1:200, Sigma-Aldrich), anti-neurofilament heavy polypeptide-NHP (rabbit, 1:500, Abcam). Notably, mouse primary antibodies were also diluted in M.O.M. protein diluent and citric buffer (10 mM) and microwave (three times for 5 min) unmasking was used for the APP staining, as previously described^47^. The next day, tissue was washed and incubated with the following secondary antibodies (1:1,000, in PBS + 0.1% Triton X-100 + 1% NGS or NDS for 1 h at room temperature): anti-mouse Alexa Fluor 405 (donkey, Thermo Fisher Scientific), anti-chicken Alexa Fluor 488 (goat or donkey, Thermo Fisher Scientific), anti-rabbit Alexa Fluor 488 (donkey, Thermo Fisher Scientific), anti-mouse Alexa Fluor 546 (donkey, Thermo Fisher Scientific), anti-goat Alexa Fluor 647 (donkey, Thermo Fisher Scientific), anti-mouse Alexa Fluor 647 (donkey, Thermo Fisher Scientific), anti-rabbit Alexa Fluor 647 (goat, Thermo Fisher Scientific) and anti-rat Alexa Fluor 647 (goat, Thermo Fisher Scientific). The tissue was counterstained with 1:10,000 DAPI in 1× PBS for 5 min, then washed and embedded with mounting medium for subsequent analysis.

Quantification of *Cx3cr1-YFP*^*creERT2*^*R26*^*tdTomato*^ specificity was performed by acquiring images on the Andor Dragonfly confocal microscope from control (78 days from TAM administration), A-EAE and C-EAE axial spinal cord sections. Data are expressed as the number (±s.e.m.) of IBA1^+^, CD3^+^, CD19^+^, GFAP^+^ being RFP^+^YFP^+^ or RFP^−^YFP^+^, or RFP^−^YFP^−^ cells from three spinal cord sections per mouse from ≥2 independent biological replicates.

SPP1^+^ microglia (RFP^+^YFP^+^) and macrophages (RFP^−^YFP^+^); NDUFS4^+^SPP1^+^ microglia (RFP^+^YFP^+^); and SPP1^+^ microglia (CX3CR1^+^) were quantified by acquiring images on the Leica microsystems CTR4000 confocal microscope (Leica Biosystems) and expressed as number of cells (±s.e.m.) from three spinal cord sections per mouse from ≥4 independent biological replicates.

Caspase-3^+^ microglia (IBA1^+^) from *Cx3cr1-YFP*^*creERT2*^*Ndufs4*^*flox/flox*^ mice and GP91-PHOX^+^ microglia (IBA1^+^), astrocytes (GFAP^+^), oligodendrocytes (OLIG2^+^) and neurons (NEUN^+^) from EAE mice that received the small-molecule treatments were quantified from *n* = 3 images of the posterior columns of spinal cord axial sections acquired through the Leica microsystems DMI4000 microscope (Leica Biosystems) and expressed as the number of cells (±s.e.m.) from ≥4 independent biological replicates.

Quantification of GP91-PHOX^+^SPP1^+^ microglia (RFP^+^YFP^+^) and macrophages (RFP^−^YFP^+^); GP91-PHOX^+^SPP1^+^ microglia (IBA1^+^); and microglial arborizations was performed by acquiring images on the Andor Dragonfly confocal microscope. Data are expressed as the number of cells (±s.e.m.) from three spinal cord sections per mouse from ≥4 independent biological replicates. Skeleton analysis was performed as described previously^41^ using Fiji v.2.0.0. software plugins AnalyzeSkeleton (2D/3D) after applying cut-off criteria = 4.0, pixel radius = 3 and mask weight = 0.6. Data are expressed as the average number of endpoints per cell, and average process length per μm^2^ from *n* ≥ 3 ROIs per mouse from *n* ≥ 3 independent biological replicates (±s.e.m.).

Human tissue immunofluorescence was performed on post mortem brain tissue sections after fixing them in 4% PFA for 10 min. After blocking with 10% NDS in 0.3% Triton X-100 PBS solution (PBS-T), consecutive sections were incubated overnight at 4 °C with primary antibodies against MHC-II (mouse, 1:500, BioLegend) and SPP1 (rabbit, 1:400, Abcam), or antibodies against MHC-II (mouse, 1:500, BioLegend) and NDUFS4 (rabbit, 1:300, Atlas Antibodies) in 1.25% NDS PBS-T. The next day, autofluorescence was quenched using TrueBlack Lipofuscin Quencher (23007, Biotium, 1:20 dilution) according to the manufacture’s recommendations. The sections were then incubated for 1 h at room temperature in 1.5% NDS PBS with the following secondary antibodies (1:250): AlexaFluor 488 donkey anti-rabbit (Thermo Fisher Scientific) and AlexaFluor 546 donkey anti-mouse (Thermo Fisher Scientific). After three PBS washes, the sections were incubated in DAPI (1:1,000) for 5 min, mounted using ProLong Diamond Antifade Mountant (P36961, Invitrogen), then imaged using a whole-slide scanner at ×20 resolution (Zeiss Axioscan Z1).

For in vitro immunocytochemistry, hiMGs were plated onto a glass coverslip (VWR) fixed with 4% PFA (Sigma-Aldrich) for 10 min and washed twice with 1× PBS. Next, cells were blocked with 10% NGS 0.1% Triton X-100 in 1× PBS for 30 min, and incubated overnight with anti-IBA1 (1:500, WAKO) and anti-CSF1R (1:100, Invitrogen) antibodies at 4 °C. The next day, cells were washed three times in 1× PBS, and incubated for 1 h with anti-rabbit Alexa 488 and anti-rat Alexa 546 secondary antibodies (1:1,000, Thermo Fisher Scientific) at room temperature. Cells were counterstained with DAPI (1:10,000, Abcam) in 1× PBS for 5 min and embedded in mounting medium (Fluoromount-G, Invitrogen). The Leica microsystems CTR4000 confocal microscope (Leica Biosystems) was used to take confocal images of the staining.

### CyTOF analysis

The first groups of *Ndufs4* WT and KO EAE mice (*n* = 4) and of small-molecule treated EAE mice (*n* = 6) for CyTOF were deeply anaesthetized with i.p. injections of ketamine (10 mg ml^−1^, Boehringer Ingelheim) and xylazine (1.17 mg ml^−1^, Bayer) and then transcardially perfused with ice-cold 1× PBS + 0.1% EDTA. The spinal cords were extracted from the spinal columns using a 5 ml syringe filled with ice-cold sorting buffer (DMEM no phenol red (Thermo Fisher Scientific) + 25 mM HEPES (Sigma-Aldrich) + 5% dialysed FBS (Thermo Fisher Scientific) + 1 mM EDTA (Honeywell Fluka) + 1× GlutaMAX (Thermo Fisher Scientific)). The spinal cords were mechanically dissociated in a glass Dounce tissue homogenizer with 7 ml of cold homogenization buffer (sorting buffer + 10 mg ml^−1^ of DNase). After tissue dissociation, the suspension was filtered through a pre-wet 40 μM strainer and the homogenizer rinsed with 2 ml of homogenization buffer. The samples were then transferred to 15 ml Falcon tubes and 2.7 ml of 90% Percoll (GE Healthcare) in 10× PBS (Thermo Fisher Scientific) was added to each sample to remove myelin and debris. The 15 ml Falcon tubes were then inverted ten times gently and the samples were centrifuged (Heraeus Megafuge 40R, Thermo Fisher Scientific) at 800*g* for 20 min at 4 °C with a brake speed of 0. Myelin debris visibly layered at the surface was carefully removed with a pipette. Ice-cold buffer (95% autoMACS rinsing solution (Miltenyi Biotec) and 5% MACS BSA (Miltenyi Biotec)) was added to the samples (to fill the 15 ml tubes) and the samples were centrifuged (Heraeus Megafuge 40R, Thermo Scientific Scientific) at 800*g* for 5 min at 4 °C to remove the remaining Percoll. Pellets were resuspended in sorting buffer (1 ml + 7 ml) and then further centrifuged at 800*g* for 5 min at 4 °C.

The cell pellet was resuspended in 100 μl of cold buffer and 10 μl of CD45 positive-selection MicroBeads (Miltenyi Biotec) were added to each sample and mixed well. The samples were incubated at 20 min on ice and gently mixed every 5 min. After incubation, 2 ml of cold buffer was added to wash the samples and the samples were then centrifuged at 300*g* for 10 min at 4 °C. After centrifugation, the supernatant was removed with a pipette, and the pellet was resuspended in 500 μl of cold buffer. The LS MACS columns (Miltenyi Biotec) were placed in the QuadroMACS Separator (Miltenyi Biotec) on the MACS MultiStand (Miltenyi Biotec) and rinsed three times with 3 ml of cold buffer. After the columns were rinsed, the 500 μl cell suspension was added onto the column. Unlabelled (that is, CD45^−^) cells from the filtrate were collected and the column was washed three times with 3 ml of cold buffer. Finally, 5 ml of cold buffer was added to the column and subsequently flushed with a plunger to isolate the CD45^+^ cells. The samples were centrifuged at 300*g* for 5 min at 4 °C. The supernatant was removed, and the cells were resuspended in 50 μl of Maxpar PBS (Fluidigm). A working solution (1:500) of 5 μM Cell-ID Cisplatin (Fluidigm) was prepared and 50 μl was added to the cells (1:1 mix) for a final concentration of 2.5 μM. The cells were incubated for 90 s and the cisplatin was quenched with 5 ml of Maxpar cell staining buffer (Fluidigm). The samples were centrifuged at 300*g* for 5 min at 4 °C, the supernatant was removed and the cells were resuspended in 1 ml of 1× Fix I buffer (Fluidigm) diluted in Maxpar PBS and left to incubate for 10 min at room temperature. The samples were centrifuged at 1,000*g* for 7 min at room temperature, the supernatant was removed and the cells were resuspended in 1 ml of 1× barcode perm buffer (Fluidigm) and then recentrifuged at 1,000*g* for 7 min at room temperature. The supernatant was removed, and the cells were resuspended in 800 μl of 1× barcode perm buffer. Then, 100 μl of a Cell-ID palladium (Pd) unique barcode (Fluidigm) was added to each sample and left to incubate for 30 min at room temperature. The samples were gently mixed after 15 min. After barcoding, the samples were centrifuged at 1,000*g* for 7 min at room temperature, the supernatant was removed, and the cells were resuspended in 2 ml of Maxpar cell staining buffer. The samples were recentrifuged at 1,000*g* for 7 min at room temperature, the supernatant was removed and the cells were resuspended in 100 μl of Maxpar cell staining buffer. All of the barcoded samples were combined into one single 5 ml polypropylene tube (BD Biosciences). Each sample tube was washed with 500 μl of Maxpar cell staining buffer, transferred to the single tube (containing the barcoded samples) and centrifuged at 1,000*g* for 7 min at room temperature. The supernatant was removed, the cells were resuspended in 50 μl of Maxpar cell staining buffer and CD16/CD32 Fc receptor block (BD Biosciences) at 1:25 was added. The sample was left to incubate for 10 min at room temperature. After 10 min, 50 μl of the surface antibody cocktail (Supplementary Data 6) was added to the sample. The sample was mixed with gentle pipetting and incubated for 15 min at room temperature. At 15 min, the sample was gently mixed and left to incubate for an additional 15 min. After surface antibody incubation, 2 ml of Maxpar cell staining buffer was added to the sample and it was centrifuged at 1,000*g* for 7 min at room temperature. The supernatant was removed, 2 ml of Maxpar cell staining buffer was added to the sample and it was recentrifuged at 1,000*g* for 7 min at room temperature. The supernatant was removed, and the cells were fixed in 1 ml of 1× Maxpar Fix I buffer for 15 min at room temperature. Then, 2 ml of Maxpar Perm-S buffer (Fluidigm) was added and the sample was centrifuged at 1,000*g* for 7 min at room temperature. The supernatant was removed, 2 ml of Maxpar Perm-S buffer was added and the sample was recentrifuged at 1,000*g* for 7 min at room temperature. The supernatant was removed (leaving behind ~50 μl) and 50 μl of the cytoplasmic/secreted protein antibody cocktail (Supplementary Data 6) was added. The sample was gently mixed with a pipette and left to incubate for 30 min at room temperature (at 15 min the sample was gently mixed). After incubation, the cells were washed with 2 ml of Maxpar cell staining buffer, and centrifuged at 1,000*g* for 7 min at room temperature. The supernatant was removed, 2 ml of Maxpar cell staining buffer was added to the sample, and it was recentrifuged at 1,000*g* for 7 min at room temperature. The supernatant was removed, and the cells were fixed in 1 ml of 1.6% PFA for 10 min at room temperature. After 10 min, the cells were centrifuged at 1,000*g* for 7 min at room temperature. The supernatant was removed, and the cells were incubated with 1 ml of Cell-ID Intercalator-IR diluted in Maxpar fix and perm buffer (1:1,000 dilution of 125 μM stock solution) (Fluidigm) for 1 h at room temperature. After incubation, the cells were centrifuged at 1,000*g* for 7 min at room temperature. The supernatant was removed, 2 ml of Maxpar cell staining buffer was added to the sample, and it was recentrifuged at 1,000*g* for 7 min at room temperature. The supernatant was removed, 1 ml of Maxpar cell staining buffer was added to the sample, and it was recentrifuged at 1,000*g* for 10 min at room temperature. At this stage, the supernatant was removed leaving between 50–100 μl of solution covering the cell pellet. The sample was stored for 48 h at 4 °C until acquisition.

The next day, the other groups of *Ndufs4* WT and KO EAE mice (*n* = 4) and of small-molecule-treated EAE mice (*n* = 6) were processed for CyTOF according to the same protocol. This set of barcoded samples was stored for 24 h at 4 °C until acquisition. On the day of acquisition, the samples were combined into a single tube of eight barcoded samples. The cells were washed in 1 ml of Maxpar Cell Acquisition Solution (Fluidigm) and centrifuged at 1,000*g* for 7 min at room temperature. The supernatant was removed, and the samples were left on ice until acquisition. Directly before acquisition, the cells were resuspended in 1 ml of 0.1× EQ Four Element Calibration Beads (Fluidigm) diluted in Maxpar Cell Acquisition Solution, passed through a 40 μM filter and then analysed on the Helios mass cytometer using a WB injector. The data files were debarcoded using Fluidigm software (v.7.0.8493) with a Mahalanobis distance of 2.50 (*Ndufs4* WT and KO EAE mice) and 8.0 (small-molecule-treated EAE mice) then concatenated and exported as FCS files.

The individual FCS files were imported into FlowJo v.10 (BD Biosciences) and live cells were selected using Boolean gating (the gating strategy is reported in Extended Data Fig. 10j). We first gated time by 140Ce^−^ beads to capture all cells. These events were then gated on time by event length followed by gating on viable cells (194 Pt^−^). We next further gated viable cells positively stained with the nucleic acid intercalator iridium (191Ir^+^193Ir^+^). Viable cells were then gated to select for myeloid cells (CD45^+^CD11b^+^) and lymphoid cells (CD45^+^CD11b^−^) and exported as individual FCS files to be further analysed in R v.4.1.3. Fcs files were loaded using flowCore (2.6.0). For the small-molecule treated EAE samples, we used the package GdClean_0.0.0.9000 (https://github.com/JunweiLiu0208/GdClean) to estimate and remove gadolinium contamination from the samples. Flow sets were then converted to single-cell experiment objects using CATALYST (v.1.18.1). Using CATALYST, cells were assigned to 1 of 100 grid points on a self-organizing map and metaclustered into 20 groups using the consensus clustering method considering cell-surface features only^87^. UMAP dimensionality reduction was performed using the CATALYST wrapper around scater runUMAP with the default parameters and data from cell-surface features only. Each of the clusters was assigned to cell types on the basis of the expression of cell-type-specific protein markers (Supplementary Data 7).

All packages are publicly available at the Comprehensive R Archive Network (https://cran.r-project.org), the Bioconductor project (http://bioconductor.org) or their respective GitHub pages. Complete R analysis workflow for CyTOF is available at GitHub (https://github.com/regan-hamel/ComplexI), and CyTOF code and data has been published on Zenodo (10.5281/zenodo.10510046).

### Statistical analyses

Unless otherwise stated, statistical analyses were performed using Graph Pad Prism 9 for macOS (GraphPad Software). EAE scores were compared using two-way ANOVA followed by Bonferroni multiple-comparisons test. Differences between two groups were analysed using unpaired *t*-tests unless otherwise stated. Differences among more than two groups were analysed using one-way ANOVA followed by Tukey’s multiple-comparison test unless otherwise stated. Data are shown in the text and figures as the mean ± s.e.m. unless otherwise stated *P* < 0.05 was considered to be significant in all analyses unless otherwise stated. Methods of the other statistical analyses have been described in the relevant sections reported above.

### Ethics statement

Animal research has been regulated under the Animals (Scientific Procedures) Act 1986 Amendment Regulations 2012 following ethical review by the University of Cambridge Animal Welfare and Ethical Review Body (AWERB). Animal work was covered by the PPL 80/2457 and PPL PP2135981 (to S.P.). Research on human tissue has been approved by the London—Queen Square Research Ethics Committee (IRAS reference: 279989) and the Cambridge University Hospitals NHS Foundation Trust Research and Development Department.

### Reporting summary

Further information on research design is available in the Nature Portfolio Reporting Summary linked to this article.

## Online content

Any methods, additional references, Nature Portfolio reporting summaries, source data, extended data, supplementary information, acknowledgements, peer review information; details of author contributions and competing interests; and statements of data and code availability are available at 10.1038/s41586-024-07167-9.

### Supplementary information

Reporting Summary

Supplementary Data 1 scRNA-seq analysis of myeloid cells isolated from *Cx3cr1-YFP*^*creERT2*^*R26*^*tdTomato*^ EAE mice. Tabs show DEGs and GO terms for each cluster.

Supplementary Data 2 scRNA-seq analysis of myeloid cells subclusters isolated from *Cx3cr1-YFP*^*creERT2*^*R26*^*tdTomato*^ EAE mice and reanalysis of human datasets. Tabs show DEGs, and GO terms for subclusters 4.1-3 and 2.0-4, numbers/percentages of MAMS and DEGs of MAMS.

Supplementary Data 3 LC–MS analysis of the intracellular metabolome of myeloid cells isolated from *Cx3cr1-YFP*^*creERT2*^*R26*^*tdTomato*^ EAE mice. Tabs report original raw data, normalized data and data depicted in the volcano plots.

Supplementary Data 4 Multivariate analysis of the LD-REIMS metabolomics data of the EAE mice. Tabs show the average normalized intensity values of metabolites of interest driving the stratification of various regions of interest. Species names have also been assigned alongside the corresponding *m*/*z* values where appropriate.

Supplementary Data 5 scRNA-seq analysis of CNS cells isolated from WT and Nd6 (control and EAE) mice. Tabs show number of cells with corresponding Fisher’s test (differences in clusters with at least 100 cells and *P*_adj_ < 0.01 were considered), DEGs and GO terms among cluster and between conditions (Nd6 versus WT).

Supplementary Data 6 scRNA-seq analysis of CNS cells isolated from *Ndufs4*-WT and *Ndufs4*-KO (Ctrl and EAE) mice. Tabs show the number of cells with corresponding Fisher’s test (differences in clusters with at least 100 cells and *P*_adj_ <  0.01 were considered), DEGs and GO terms among cluster and between conditions (*Ndufs4*-KO versus *Ndufs4* WT).

Supplementary Data 7 CyTOF panel, antibodies and cell markers used for cell assignment.

### Source data

Source Data Fig. 1

Source Data Fig. 2

Source Data Fig. 3

Source Data Fig. 4

Source Data Extended Data Fig. 1

Source Data Extended Data Fig. 2

Source Data Extended Data Fig. 7

Source Data Extended Data Fig. 8

Source Data Extended Data Fig. 9

Source Data Extended Data Fig. 10

Source Data Extended Data Fig. 11

## Data Availability

The bulk and single-cell RNA-seq datasets are publicly available in raw (fastq) and processed (expression matrix) format at the Gene Expression Omnibus (GSE248175). The processed data can be explored as Shiny Apps (https://bioinf.stemcells.cam.ac.uk/shiny/pluchino/complex1_microglia/shinyapp_cremato/; https://bioinf.stemcells.cam.ac.uk/shiny/pluchino/complex1_microglia/shinyapp_schirmer/; https://bioinf.stemcells.cam.ac.uk/shiny/pluchino/complex1_microglia/shinyapp_absinta/; https://bioinf.stemcells.cam.ac.uk/shiny/pluchino/complex1_microglia/shinyapp_nd6/; https://bioinf.stemcells.cam.ac.uk/shiny/pluchino/complex1_microglia/shinyapp_ndufs4/; https://bioinf.stemcells.cam.ac.uk/shiny/pluchino/complex1_microglia/bulkanalyser/). Metabolomics data are provided in Supplementary Data 3 (LC–MS) and Supplementary Data 4 (LD-REIMS). The CyTOF dataset is available online (https://data.mendeley.com/v1/datasets/w5wtx43528/draft?a=d3079d50-32aa-43d4-b719-c196d8af220e) and at Zenodo (https://zenodo.org/records/10510047). Source data are provided with this paper.

## Code availability

The R analysis workflow for the scRNA-seq data is available at GitHub (https://github.com/Core-Bioinformatics/complex1_microglia/tree/) and Zenodo (https://zenodo.org/records/10519730).
