# Supplementary material for: Mitochondrial complex I activity in microglia sustains neuroinflammation
Source: Nature. 2024 Mar 13;628(8006):195–203. doi: 10.1038/s41586-024-07167-9 (PMC10990929; doi:10.1038/s41586-024-07167-9)
Supplement: Supplementary file 1 — Reporting Summary [file 41586_2024_7167_MOESM1_ESM.pdf]

nature portfolio

Corresponding author(s): Peruzzotti-Jametti Luca
Pluchino Stefano

Last updated by author(s): Jan 10, 2024

Reporting Summary

Nature Portfolio wishes to improve the reproducibility of the work that we publish. This form provides structure for consistency and transparency in reporting. For further information on Nature Portfolio policies, see our Editorial Policies and the Editorial Policy Checklist.

Statistics

For all statistical analyses, confirm that the following items are present in the figure legend, table legend, main text, or Methods section.

n/a	Confirmed	
☐	☒	The exact sample size (*n*) for each experimental group/condition, given as a discrete number and unit of measurement
☐	☒	A statement on whether measurements were taken from distinct samples or whether the same sample was measured repeatedly
☐	☒	The statistical test(s) used AND whether they are one- or two-sided<br>*Only common tests should be described solely by name; describe more complex techniques in the Methods section.*
☐	☒	A description of all covariates tested
☐	☒	A description of any assumptions or corrections, such as tests of normality and adjustment for multiple comparisons
☐	☒	A full description of the statistical parameters including central tendency (e.g. means) or other basic estimates (e.g. regression coefficient) AND variation (e.g. standard deviation) or associated estimates of uncertainty (e.g. confidence intervals)
☐	☒	For null hypothesis testing, the test statistic (e.g. *F*, *t*, *r*) with confidence intervals, effect sizes, degrees of freedom and *P* value noted<br>*Give P values as exact values whenever suitable.*
☒	☐	For Bayesian analysis, information on the choice of priors and Markov chain Monte Carlo settings
☐	☒	For hierarchical and complex designs, identification of the appropriate level for tests and full reporting of outcomes
☐	☒	Estimates of effect sizes (e.g. Cohen's *d*, Pearson's *r*), indicating how they were calculated

Our web collection on statistics for biologists contains articles on many of the points above.

Software and code

Policy information about availability of computer code

Data collection	See Methods.
Data analysis	The R analysis workflow for the scRNAseq data is available on GitHub (https://github.com/Core-Bioinformatics/complex1_microglia/tree/) and Zenodo (https://zenodo.org/records/10519730). All packages used throughout the analysis are listed in the header of the scripts. The analyses and benchmarking were performed on dedicated Linux servers (Debian GNU/Linux v10 and Linux kernel version 4.19.0-12-amd64).<br>The CyTOF-related packages were CATALYST, flowCore, and we used the package GdClean (https://github.com/JunweiLiu0208/GdClean) to estimate and remove gadolinium contamination from samples. The complete R analysis workflow for the CyTOF is deposited on GitHub (https://github.com/regan-hamel/ComplexI). The CyTOF code and data has been published on Zenodo (https://zenodo.org/records/10510047). The version numbers of the software/packages/tools are: R version 4.1.3 (2022-03-10); Platform: x86_64-apple-darwin17.0 (64-bit); Running under: macOS 14.1; SeuratObject_4.1.3; Seurat_4.3.0; scater_1.22.0; scuttle_1.4.0; CATALYST_1.18.1; SingleCellExperiment_1.16.0 SummarizedExperiment_1.24.0 Biobase_2.54.0; GenomicRanges_1.46.1; GenomeInfoDb_1.30.1; IRanges_2.28.0; S4Vectors_0.32.4; BiocGenerics_0.40.0; MatrixGenerics_1.6.0; matrixStats_0.63.0; readxl_1.4.1; ggpubr_0.5.0; mgcv_1.8-41; nlme_3.1-161; ggplot2_3.4.0; pheatmap_1.0.12; RColorBrewer_1.1-3; dplyr_1.0.10; stringr_1.5.0; flowCore_2.6.0; GdClean_0.0.0.9000.<br>Analysis of LC-MS data was performed with MetaboAnalyst 5.0 (https://www.metaboanalyst.ca/) and Graph Pad Prism 9 for macOS, (GraphPad Software, San Diego, CA, USA, www.graphpad.com). Images were processed with Fiji 2.0.0. software, and all remaining statistics were performed using Graph Pad Prism 9 for macOS. FACS data were processed with the FACSDiva Software version 9.0.1 and FlowJo v10 (BD Biosciences).

For manuscripts utilizing custom algorithms or software that are central to the research but not yet described in published literature, software must be made available to editors and reviewers. We strongly encourage code deposition in a community repository (e.g. GitHub). See the Nature Portfolio guidelines for submitting code & software for further information.

Data

Policy information about availability of data

All manuscripts must include a data availability statement. This statement should provide the following information, where applicable:

- Accession codes, unique identifiers, or web links for publicly available datasets
- A description of any restrictions on data availability
- For clinical datasets or third party data, please ensure that the statement adheres to our policy

The bulk and single-cell RNAseq datasets are publicly available, in raw (fastq) and processed (expression matrix) format, on the Gene Expression Omnibus (GEO), accession GSE248175. The R analysis workflow for the scRNAseq data is available on GitHub (https://github.com/Core-Bioinformatics/complex1_microglia/tree/) and Zenodo (https://zenodo.org/records/10519730).
The processed data can be further explored as Shiny Apps:
https://bioinf.stemcells.cam.ac.uk/shiny/pluchino/complex1_microglia/shinyapp_cremato/
https://bioinf.stemcells.cam.ac.uk/shiny/pluchino/complex1_microglia/shinyapp_schirmer/
https://bioinf.stemcells.cam.ac.uk/shiny/pluchino/complex1_microglia/shinyapp_absinta/
https://bioinf.stemcells.cam.ac.uk/shiny/pluchino/complex1_microglia/shinyapp_nd6/
https://bioinf.stemcells.cam.ac.uk/shiny/pluchino/complex1_microglia/shinyapp_ndufs4/
https://bioinf.stemcells.cam.ac.uk/shiny/pluchino/complex1_microglia/bulkanalyser/
Metabolomics data are reported in 'Supplementary Data 3' for LC-MS and 'Supplementary Data 4' for LD-REIMS. The CyTOF dataset is available (https://data.mendeley.com/v1/datasets/w5wtx43528/draft?a=d3079d50-32aa-43d4-b719-c196d8af220e) and Zenodo (https://zenodo.org/records/10510047). The R analysis workflow for the scRNAseq data is available on GitHub (Core-Bioinformatics/complex1_microglia).

Research involving human participants, their data, or biological material

Policy information about studies with human participants or human data. See also policy information about sex, gender (identity/presentation), and sexual orientation and race, ethnicity and racism.

Reporting on sex and gender	We used post-mortem samples from one male SP-MS patient (age: 45 years old) obtained from the Multiple Sclerosis and Parkinson´s Tissue Bank (Imperial College London).
Reporting on race, ethnicity, or other socially relevant groupings	No further information is available.
Population characteristics	Secondary progressive MS patient (male, 45 years old).
Recruitment	Samples were obtained from the Multiple Sclerosis and Parkinson´s Tissue Bank (Imperial College London).
Ethics oversight	Research on human tissue has been approved by the London - Queen Square Research Ethics Committee (IRAS reference: 279989) and the Cambridge University Hospitals NHS Foundation Trust Research and Development Department.

Note that full information on the approval of the study protocol must also be provided in the manuscript.

Field-specific reporting

Please select the one below that is the best fit for your research. If you are not sure, read the appropriate sections before making your selection.

☒ Life sciences ☐ Behavioural & social sciences ☐ Ecological, evolutionary & environmental sciences

For a reference copy of the document with all sections, see nature.com/documents/nr-reporting-summary-flat.pdf

Life sciences study design

All studies must disclose on these points even when the disclosure is negative.

Sample size	Data from published (Peruzzotti-Jametti et al. Cell Stem Cell 2018 and Peruzzotti-Jametti et al. Plos Biology 2021) and preliminary work has been used to obtain estimates of standard deviation (SD) and population distribution. Sample size calculations were carried out for an effect size of 0.5, 80% power, 5% level of significance, and for highest SD of the outcomes assessed.
Data exclusions	We excluded cells from scRNAseq samples based on quality control criteria, which are thoroughly described in the Methods. The R scripts used for the exclusion of low quality cells are available. We excluded low quality and gadolinium contaminated cells from the CyTOF analysis using criteria described in the Methods.
Replication	EAE behavioral data are derived from at least 2 independent EAE experiments (Extended Data Figure 11c-e are derived from 1 EAE experiment) from which tissues were isolated for downstream ex vivo analyses. All in vitro experiments were repeated at least twice except for the cytotoxicity experiments, the mtROS experiments of Extended Data Figure 11a-b, the Extended Data Figure 7l and Extended Data Figure 8i.

Randomization	For tamoxifen and small molecule treatments of EAE mice, mice were randomly assigned to each treatment group on the first day of treatment (one week after onset) so that the mean baseline EAE score of each group was not statistically different. In all other occasions, mice and samples were randomly selected by blind assessors.
Blinding	Investigators assessing in vivo (e.g., behavioural), ex vivo (e.g., pathological), and in vitro (e.g., cell cultures) outcomes were blinded to group allocation.

Reporting for specific materials, systems and methods

We require information from authors about some types of materials, experimental systems and methods used in many studies. Here, indicate whether each material, system or method listed is relevant to your study. If you are not sure if a list item applies to your research, read the appropriate section before selecting a response.

Materials & experimental systems

n/a	Involved in the study	
☐	☒	Antibodies
☐	☒	Eukaryotic cell lines
☒	☐	Palaeontology and archaeology
☐	☒	Animals and other organisms
☒	☐	Clinical data
☒	☐	Dual use research of concern
☒	☐	Plants

Methods

n/a	Involved in the study	
☒	☐	ChIP-seq
☐	☒	Flow cytometry
☒	☐	MRI-based neuroimaging

Antibodies

Antibodies used

The following primary antibodies were used:
anti-RFP antibody (rabbit, 1:400, Abcam, ab62341),
anti-YFP/GFP antibody (chicken, 1:1000, Abcam, ab13970),
anti-CD3 (rat, 1:500, eBioscience, 14-0032-82),
anti-CD19 (rabbit, 1:500, Abcam, ab245235),
anti-GFAP (mouse, 1:400, Sigma-Aldrich, MAB360)
anti-NEUN (mouse, 1:400, Sigma-Aldrich, MAB377),
anti-OLIG2 (goat, 1:500, Novus biologicals, AF2418),
anti-gp91-phox antibody (mouse, 53/gp91[phox] 1:200, BD Biosciences, 611415; or rabbit, 1:500, Proteintech, 19013-1-AP),
anti-IBA1 (goat, 1:500, FUJIFILM Wako, 019-19741),
anti-CX3CR1 (rabbit, 1:300, Alomone labs, ACR-058),
anti-NDUFS4 (rabbit, EP7832/ab137064, 1:100, Abcam or rabbit, NBP1-31465, 1:500, Novus biologicals. For human pathology: rabbit, 1:300, Atlas Antibodies, HPA003884),
anti-CASPASE3 (mouse, 1:600, Novus biologicals, NB100-56708),
anti-SPP1 (goat, 1:500, R&D Systems, AF808. For human pathology: rabbit, 1:400, Abcam, ab8448),
anti-amyloid precursor protein-APP (mouse, 22C11, 1:200, Sigma-Aldrich, MAB348),
anti-neurofilament heavy polypeptide-NHP (rabbit, 1:500, Abcam, ab8135),
anti-MHC-II (For human pathology: mouse, 1:500, BioLegend, 327002),
anti-PLP (mouse, 1:200, BioRad, MCA839G),
anti-CSF1R (rat, 1:100, Invitrogen (AFS98) eBioscience, AB_467428),
anti-CD11b FITC (rat, 1uL per 1M cells, Clone M1/70, 553310 BD Pharmingen),
anti-CD45 BV510 (rat, 1uL per 1M cells, 30-F11, 563891 BD Horizon),
anti-CX3CR1 PE (mouse, 1uL per 1M cells, clone: SA011F11, 149005 BioLegend).

The following secondary antibodies were used:
biotinylated anti-rabbit (goat, 1:1000, Abcam, ab6720),
biotinylated anti-chicken (goat, 1:1000, Vector Laboratories, BA-9010),
biotinylated anti-mouse (goat, 1:1000, Thermo Fisher Scientific, 31800),
anti-mouse Alexa Fluor 405 (donkey, 1:1000, Thermo-Fisher, A48257),
anti-chicken Alexa Fluor 488 (goat or donkey, 1:1000, Thermo-Fisher, A78948),
anti-rabbit Alexa Fluor 488 (donkey, 1:1000, Thermo-Fisher, A21206),
anti-mouse Alexa Fluor 546 (donkey, 1:1000, Thermo-Fisher, A10036),
anti-goat Alexa Fluor 647 (donkey, 1:1000, Thermo-Fisher, A21447),
anti-mouse Alexa Fluor 647 (donkey, 1:1000, Thermo-Fisher, A31571),
anti-rabbit Alexa Fluor 647 (goat, 1:1000, Thermo-Fisher, A21244),
anti-rat Alexa Fluor 647 (goat, 1:1000, Thermo-Fisher, A21247).

For CyTOF antibodies see 'Supplementary Data 7'.

Validation

Primary and secondary antibodies were validated via immunostaining of tissues/areas with known positive expression patterns of the target protein (positive control) and compared to the signal of tissues/areas with negative expression and/or stained with secondary antibodies only (negative controls).

Eukaryotic cell lines

Policy information about cell lines and Sex and Gender in Research

Cell line source(s)	The human iPSC line (HPSI0214i-wibj_2) was obtained from Cambridge BioResource and gifted by Alessandra Granata (University of Cambridge).<br>BV2 cells were gifted by Aviva Tolkovsky (University of Cambridge) and are akin to immortalized mouse microglia cells (CSC-I2227Z Creative Bioarray).<br>SH-SY5Y cells were gifted by Michael Whitehead (University of Cambridge) and are akin to human neuroblastoma cells (CRL-2266 ATCC).<br>Primary microglia were obtained following the protocol described in the Methods.
Authentication	None of the cell lines were authenticated.
Mycoplasma contamination	Cell lines were tested for mycoplasma contamination on a regular basis using a PCR-based test and were found to be negative for mycoplasma.
Commonly misidentified lines<br>(See ICLAC register)	None.

Animals and other research organisms

Policy information about studies involving animals; ARRIVE guidelines recommended for reporting animal research, and Sex and Gender in Research

Laboratory animals	We used a Cx3cr1YFPCreERT2:R26tdTomato fate mapping mouse that was generated by crossing B6.129P2(Cg)-Cx3cr1tm2.1(cre/ERT2)Litt/WganJ with B6.Cg-Gt(ROSA)26Sortm9(CAG-tdTomato)Hze/J)55, as previously described. ND6-P25L mice were obtained from Professor Douglas Wallace (University of Pennsylvania, USA). The Cx3cr1YFPCreERT2:Ndufs4flox/flox mice were generated by crossing B6.129P2(Cg)-Cx3cr1tm2.1(cre/ERT2)Litt/WganJ with mice with conditional alleles of the Ndufs4 gene (exon 2 flanked by loxP sites). WT C57BL/6 mice were purchased from Charles River. EAE induction was performed on mice aged 8-20 weeks.
Wild animals	The study did not involve wild animals.
Reporting on sex	All EAEs were induced on female mice as described in the Methods.<br>Primary microglia were derived from unsexed pups as described in the Methods.<br>Healthy controls included both female and male mice.
Field-collected samples	The study did not involve samples collected from the field.
Ethics oversight	Animal research has been regulated under the Animals (Scientific Procedures) Act 1986 Amendment Regulations 2012 following ethical review by the University of Cambridge Animal Welfare and Ethical Review Body (AWERB). Animal work was covered by the PPL 80/2457 and PPL PP2135981 and PP4301560.

Note that full information on the approval of the study protocol must also be provided in the manuscript.

Plants

Seed stocks	n/a
Novel plant genotypes	n/a
Authentication	n/a

Flow Cytometry

Plots

Confirm that:

☒ The axis labels state the marker and fluorochrome used (e.g. CD4-FITC).

☒ The axis scales are clearly visible. Include numbers along axes only for bottom left plot of group (a 'group' is an analysis of identical markers).

☒ All plots are contour plots with outliers or pseudocolor plots.

☒ A numerical value for number of cells or percentage (with statistics) is provided.

Methodology

Sample preparation	See Methods for full details.<br>For scRNAseq the cell pellet was resuspended in 200 µL of FACS buffer and 7-AAD live/dead stain (1:50) added. The samples were sorted using 3-way purity with a 100 µM nozzle at 20 psi. The cells were collected in 1x PBS.<br>For LC-MS analysis and metabolic flux analysis with Seahorse, the cell pellet was resuspended in 100 µL of sorting buffer and SYTOX blue nucleic acid stain (0.5%) added. The samples were sorted using a BD FACS Aria III set to yield with a 85 µM nozzle.<br>For ex vivo ROS production of Cx3cr1YFPCreERT2:R26tdTomato mice was obtained after exposing CNS homogenates to 1?M rotenone or vehicle solution for 30 min, at 37 oC with constant shaking at 500 rpm. Then we stained the samples using 5?M of CellROX Deep Red Flow Cytometry Assay Kit (Thermo Fisher Scientific) and live cells analysed (SYTOX blue nucleic acid stain).<br>Ex vivo mitochondrial membrane potential of Cx3cr1YFPCreERT2:R26tdTomato mice was obtained by staining brain and spinal cord homogenates using 200 nM of the mitochondrial membrane potential indicator MitoView633 (Biotium) in sorting buffer for 30 min, at 37 oC with constant shaking at 500 rpm, and live cells analysed (SYTOX blue nucleic acid stain).<br>Ex vivo mitochondrial membrane potential of CD45+Cd11b+ myeloid cells from WT and Nd6 mice were obtained by staining brain and spinal cord homogenates on ice in sorting buffer using Brilliant Violet 510-conjugated antibody against CD45 (BD Horizon, 1 µL per 1M cells), FITC-conjugated antibody against CD11b (BD Pharmingen, 1 µL per 1M cells) and Zombie Violet Red (BioLegend, 1:500). Live cells were identified by Zombie Red (Thermo Fisher, S34857) staining. Analysis was performed using FlowJo v10 (BD Biosciences) in the Zombie Violet negative, FITC positive and Brilliant Violet 510 positive population.<br>For the analysis of superoxide production using MitoSOX, the in vitro cell pellet was resuspended in MitoSOX Red (1 µL of a 5 mM stock into 1 mL of 1x PBS) and incubated at 37C on a shaker set to 500 RPM. The cells were then spun down for 5 min 300g at RT, and the pellet was resuspended in 300 µL of 1x PBS. 10 mins prior to acquisition, DAPI was added to the sample.
Instrument	BD FACS Aria III and BD LSRFortessa
Software	FACSDiva Software version 9.0.1 and FlowJo v10 (BD Biosciences)
Cell population abundance	For scRNAseq cells were sorted using a BD FACS Aria III cell sorter set to 3-way purity.<br>Original fcs files and details on the abundance of relevant cell populations (number of cells or percentage) are available upon request.
Gating strategy	FACS sequential gating/sorting strategies are provided in the supplementary or main figures, as follows:<br>The gating strategy for Fig1 and Fig2 (isolation of cells from Cx3cr1YFPCreERT2:R26tdTomato fate mapping mouse for downstream analyses) has been provided in the Extended Data Figure 1b and referenced in the methods.<br>The gating strategy for Fig 3h (isolation of CD45+CD11b+ cells from WT and Nd6 mice) has been provided in the Extended Data Figure 9g and referenced in the methods.<br>The gating strategy for Extended Data Fig 7b (ROS production from microglia in vitro) is reported in the same panel and referenced in the methods.<br>The gating strategy for Extended Data Fig 10c (NDUFS4 protein expression) has been provided in the same panel and referenced in the methods.<br>The CyTOF gating strategy for Fig 4 and Extended Data Fig 10-11 has been provided in the Extended Data Fig 10j, Supplementary Data 7, and referenced in the methods.

☒ Tick this box to confirm that a figure exemplifying the gating strategy is provided in the Supplementary Information.
